# pH-responsive regulation of multiphase coacervate wetting *via* phase selective enrichment of fatty acids

**DOI:** 10.1039/d5sc07783d

**Published:** 2025-12-11

**Authors:** Preeti Sharma, Pankaj Singh Patwal, B. V. V. S. Pavan Kumar

**Affiliations:** a Dynamic Colloidal Systems Laboratory, Department of Chemistry, Indian Institute of Technology Roorkee Roorkee-247667 India pavan.bosukonda@cy.iitr.ac.in

## Abstract

Biomolecular condensates with multiphasic architectures organize specific biomolecular processes in different compartments and dynamically reconfigure their structure to regulate their biological functions. Here, we employ multiphase coacervates as model condensates to illustrate pH-responsive dynamic reconfiguration of multiphase wetting interactions mediated by phase selective enrichment of fatty acids. We noted that unsaturated fatty acids such as linolenic acid (LA) can enrich within specific coacervates, *via* spontaneous substitution of like charged coacervate components, to drastically alter coacervate properties such as viscosity and surface tension. By selectively enriching fatty acids within the outer phase of a multiphase coacervate, the changes in coacervate properties were used to trigger the outer phase to dewet the inner phase and separate into two droplets. Dynamic switching between wetting and dewetting states of multiphase droplets was achieved by adjusting the outer phase composition *via* pH changes, which impacted LA's ability to substitute coacervate components. Finally, chemical signaling mediated reconfiguration of coacervate-based synthetic cells was shown using urease containing microgels, which secreted pH-based chemical signals to propagate a reconfiguration front within multiphase droplet populations. Taken together, our results highlight opportunities for the design of dynamically reconfigurable synthetic cells capable of transducing chemical signals into morphological changes and suggest that lipids enriched within condensates may be involved in regulating their morphology and function.

## Abstract 2

Selective enrichment of pH-responsive fatty acids within the outer phase of multiphase coacervates controls multiphase wetting interactions by regulating interfacial tension between the two coacervate phases and surface tension of the outer phase.

## Graphical abstract

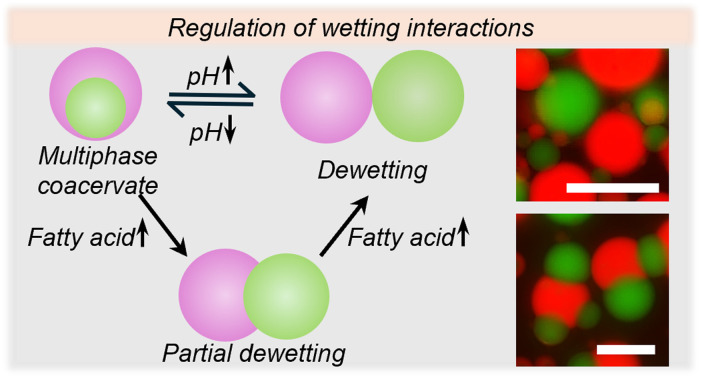

## Introduction

Biomolecular condensates are membraneless organelles formed by liquid–liquid phase separation found in living cells, playing important roles in subcellular organization,^1–3^ buffering cellular noise,^4^ macromolecular folding^5,6^ and regulation of biochemical reactions.^7,8^ Recently, condensates exhibiting multiphasic architectures have been noted, such as, nucleolus^9,10^ and stress granules,^11^ and their structural reconfiguration has been found crucial in regulation of transcriptional activity,^9^ stress response^11^ and mRNA signalling.^12^ Coacervate microdroplets serve as synthetic mimics^13–16^ of condensates to understand their roles in different aspects of cellular biology. They have also become increasingly popular as cytomimetic models due to their unique properties such as stimuli responsive phase separation,^17–21^ sequestration of small molecules^17,22,23^ and macromolecules,^24^ viscous environments,^25^ modulation of reaction rates,^26–28^ and dynamic reconfigurability.^29^ In the past few years, several multiphasic coacervate systems^30–33^ have been reported to show behaviours such as modulation of chemical equilibria,^34^ compartment content coupling,^35^ directionality of reaction cascades,^36,37^ self-sorting droplet networks,^38,39^ and dynamic phase transitions.^40,41^ The formation of these multiphasic architectures has been found to be regulated by micropolarity changes^42^ and balance of homotypic and heterotypic interactions in condensates.^43,44^ In designer nucleic acid-based droplets, the facile control of these homotypic and heterotypic interactions allowed the programming of complex behaviours such as temporal control of droplet division,^45^ and tuneable phase mixing.^46,47^ A few reports of reconfiguration of multiphase wetting have recently been shown *via* controlling phase composition,^43,48^ macromolecular crowding^49,50^ and adsorption of interfacial proteins.^51–53^ However, dynamic control of these multiphase wetting interactions remains a considerable challenge. Recent reports of small molecule mediated modulation of the microenvironment to control phase miscibility within condensates^54,55^ and their material properties^56^ suggest that small molecules could potentially also regulate multiphase wetting interactions. In particular, the recent study by Jaffrey *et al.*, highlighting the role of phospholipid partitioning into nuclear condensates in regulating condensate composition and morphology,^57^ suggested that more primitive lipids such as fatty acids could play an important role in regulating condensate properties.

**Scheme 1:**
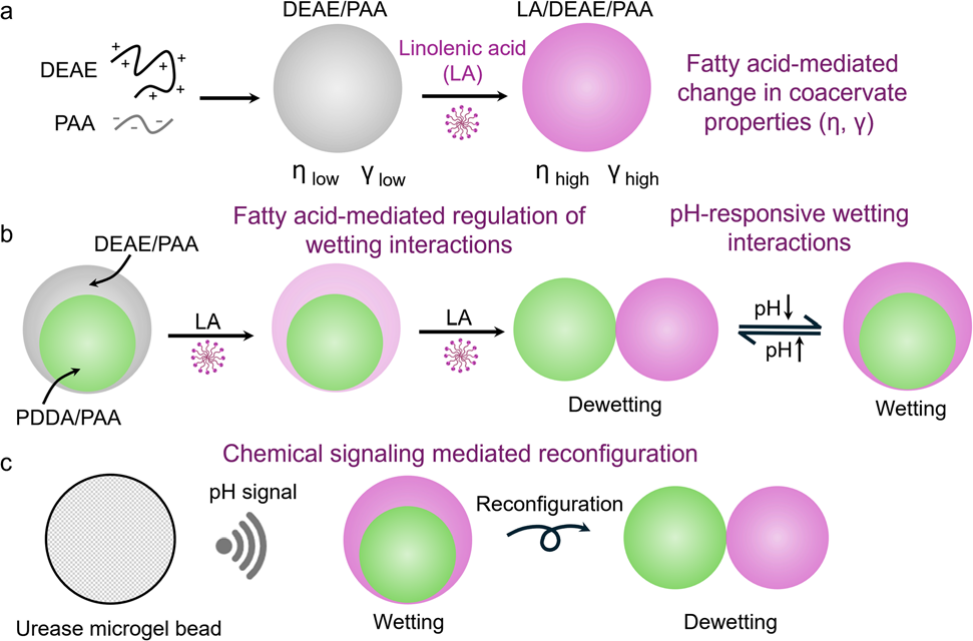
Schematic diagram showing (a) changes in properties of DEAE/PAA coacervates (η, γ) upon addition of linolenic acid (LA). (b) The selective enrichment of LA in the outer DEAE/PAA phase of a multiphase droplet regulating the multiphase wetting interactions in a concentration dependent manner and reversibly in response to changes in pH around the p*K*_a_ of LA. (c) Urease microgel beads releasing pH-based chemical signals to reconfigure the wetting interactions in multiphase coacervate droplets.

In this work, we describe the first report of pH-responsive dynamic reconfiguration of multiphase coacervate droplets caused by small molecule triggered selective change in material properties of the outer phase (Scheme 1). We noted that the addition of an unsaturated fatty acid (linolenic acid, LA) to complex coacervates composed of diethylaminoethyl-dextran hydrochloride (DEAE) and polyacrylic acid (PAA), led to spontaneous and gradual substitution of PAA from the coacervate, causing a 1.5× increase in surface tension upon partial substitution, and a two-order increase in its viscosity upon complete substitution (Scheme 1a). The substitution of PAA with LA was controlled by concentration of LA added and the charged state of LA, which depended on the pH of the environment. So, by constituting a multiphase coacervate droplet with an outer phase of LA/DEAE/PAA, we could cause a compositional shift in the outer phase *via* changes in concentration of LA and the pH of the environment to regulate the wetting interactions with the inner phase droplet (Scheme 1b). Chemical signaling mediated reconfiguration of multiphase droplets was also shown using urease containing microgels (Scheme 1c).

## Results and discussion

### Regulation of coacervate material properties *via* fatty acid enrichment

**Fig. 1:**
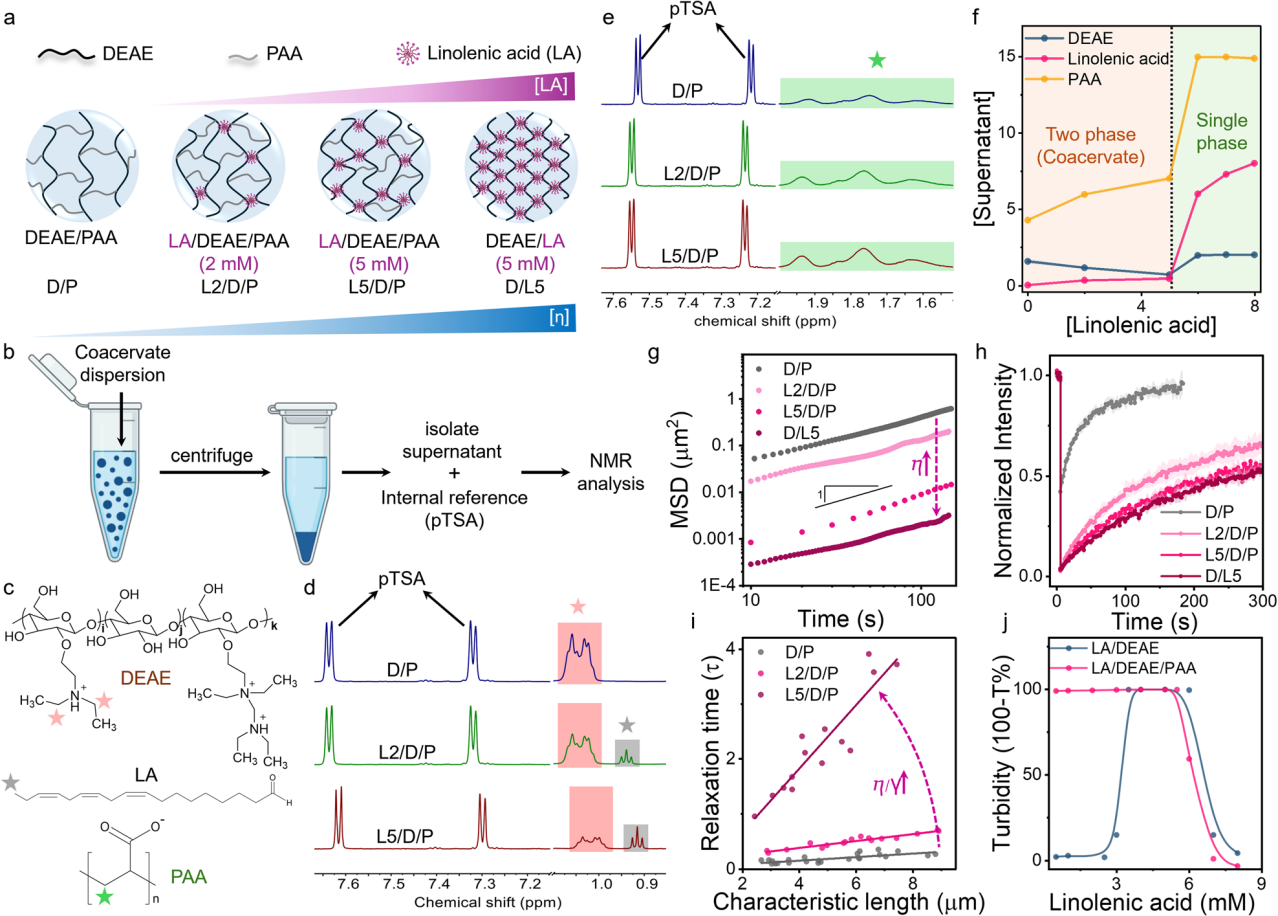
Schemes showing (a) the increase in viscosity of DEAE/PAA coacervates upon gradual substitution of PAA with LA micelles and (b) the sample preparation steps for 1H NMR analysis of the supernatant phase for tracking LA mediated changes in coacervate composition. 1H NMR data of the supernatant phase of DEAE/PAA coacervates upon addition of increasing amounts of LA showing (d) peaks corresponding to DEAE (pink box) and LA (grey box) and (e) peaks corresponding to PAA (green box) in the acidified supernatant phase. The protons chosen for 1H NMR based quantification of DEAE, LA and PAA are shown in (c). (f) Plot showing the concentration of different coacervate components in the dilute phase upon increasing LA concentration. The light brown shaded region shows the two-phase regime, and the green shaded region shows the single-phase regime with soluble polyelectrolyte complexes. (g) Mean squared displacement (MSD) of 1 µm microspheres within DEAE/PAA coacervate phases of increasing LA content. Plots of (h) fluorescence recovery after photobleaching (FRAP) curves of RITC-c-BSA and (i) droplet relaxation times *vs.* characteristic length recorded on DEAE/PAA coacervates with increasing LA content. (j) Turbidity plots showing stoichiometry of formation of LA/DEAE coacervates upon addition of LA and dissolution of DEAE/PAA coacervates above the stoichiometry of LA/DEAE coacervates *via* formation of soluble polyelectrolyte complexes.

The addition of unsaturated fatty acids such as LA to DEAE/PAA (2.4/17.3 mM; expressed as monomer concentration) coacervates above its critical micelle concentration ([LA]_cmc_ = 1 mM; Fig. S1 and S2) and above its p*K*_a_ (8.3 for self-assembled state of LA;^58^ Fig. S3) led to gradual displacement of PAA by LA to form LA/DEAE/PAA coacervates (Fig. 1a). This was studied by the analysis of 1H NMR peak areas for selected protons corresponding to each of the 3 coacervate components (LA, DEAE, and PAA) present in the supernatant phase (Fig. 1c and Note S1). The coacervate dispersion was prepared in D_2_O, and the supernatant phase was isolated by centrifugation, followed by addition of a known amount of an internal reference (*p*-toluenesulfonic acid, pTSA) for peak area calibration (Fig. 1b). It is important to note that for quantification of PAA, the supernatant was separately acidified to avoid overlap of NMR peaks with those corresponding to DEAE (Note S1). The 1H NMR analysis showed that for 2 mM and 5 mM LA added, the peak areas for the protons corresponding to DEAE decreased while those corresponding to PAA (in acidified supernatant) increased (Fig. 1d and e). This indicated that, as increasing amounts of LA were added, the LA and DEAE content within coacervates increased while the PAA content decreased (Fig. 1f). Furthermore, it suggested that stronger interactions of LA micelles with DEAE displaced PAA from the coacervate.

The rise in LA content of the coacervates was also followed *via* an increase in red fluorescence intensity of Nile Red staining the LA assemblies within the coacervate droplets (Fig. S4). The emission spectra of Nile Red in the DEAE/PAA phase containing LA (2.5 mM) at pH 9 were the same as its emission when present in LA micelles, indicating that LA micelles did not significantly transform to higher order assemblies within the DEAE/PAA phase due to ion pairing interactions leading to a decrease in head group areas. This was also confirmed by small-angle X-ray scattering (SAXS) data of the DEAE/PAA coacervate phase containing LA, which showed a broad Bragg peak at 1.20 nm^−1^, suggesting weak ordering of LA micelles in the coacervate phase, and notably the peak intensity increased with concentration of LA and was completely absent in the native DEAE/PAA phase (Fig. S5 and S6). Turbidity studies showed that DEAE/PAA coacervates dissolved upon addition of LA (6 mM) beyond the 1 : 2 (DEAE : LA) stoichiometry, forming soluble polyelectrolyte–fatty acid complexes, which was consistent with the narrow 1 : 2 stoichiometry range for the formation of DEAE/LA coacervates (Fig. 1j, S7 and S8).

Interestingly, microrheology studies revealed a two-order increase in viscosity on going from DEAE/PAA to DEAE/LA coacervates, showing viscosities of 0.8 Pa.s and 88.2 Pa.s, respectively, which is unprecedented for small molecule mediated changes in coacervate properties (Fig. 1a and g). Droplet fusion dynamics studies showed that the increasing LA content of the coacervates led to slower droplet relaxation times, with LA/DEAE/PAA (5/2.5/25 mM) showing over nine times the relaxation time of DEAE/PAA droplets. The inverse capillary velocity (η/γ) values also increased with a rise in LA content, showing values of 0.032 s µm^−1^, 0.063 s µm^−1^ and 0.578 s µm^−1^ when 0 mM, 2 mM and 5 mM LA were added to DEAE/PAA, respectively (Fig. 1i). Using the viscosity values obtained from microrheology and inverse capillary velocity values from droplet coalescence studies, surface tension values were found to be 25 µN m^−1^, 20.63 µN m^−1^ and 29.04 µN m^−1^ for DEAE/PAA with 0 mM, 2 mM and 5 mM of LA added, respectively. The increase in surface tension was also consistent with the decrease in water content of the coacervate phase from 86% for DEAE/PAA to 70% for LA/DEAE/PAA (5/2.5/25 mM) (Table S2). Fluorescence recovery after photobleaching studies on coacervate droplets containing RITC labelled cationized BSA (RITC-c-BSA) revealed an 8× increase in fluorescence recovery half-time (*τ*_1/2_) for DEAE/PAA and DEAE/LA coacervates, showing *τ*_1/2_ values of 45 s and 365 s, suggesting slower dynamics due to a rise in viscosity and increased interaction with coacervate components (Fig. 1h and Table S1). The above results indicate that small molecules such as linolenic acid are able to drastically modulate coacervate material properties.

### Fatty acid-mediated control of interfacial tension in multiphase droplets

**Fig. 2:**
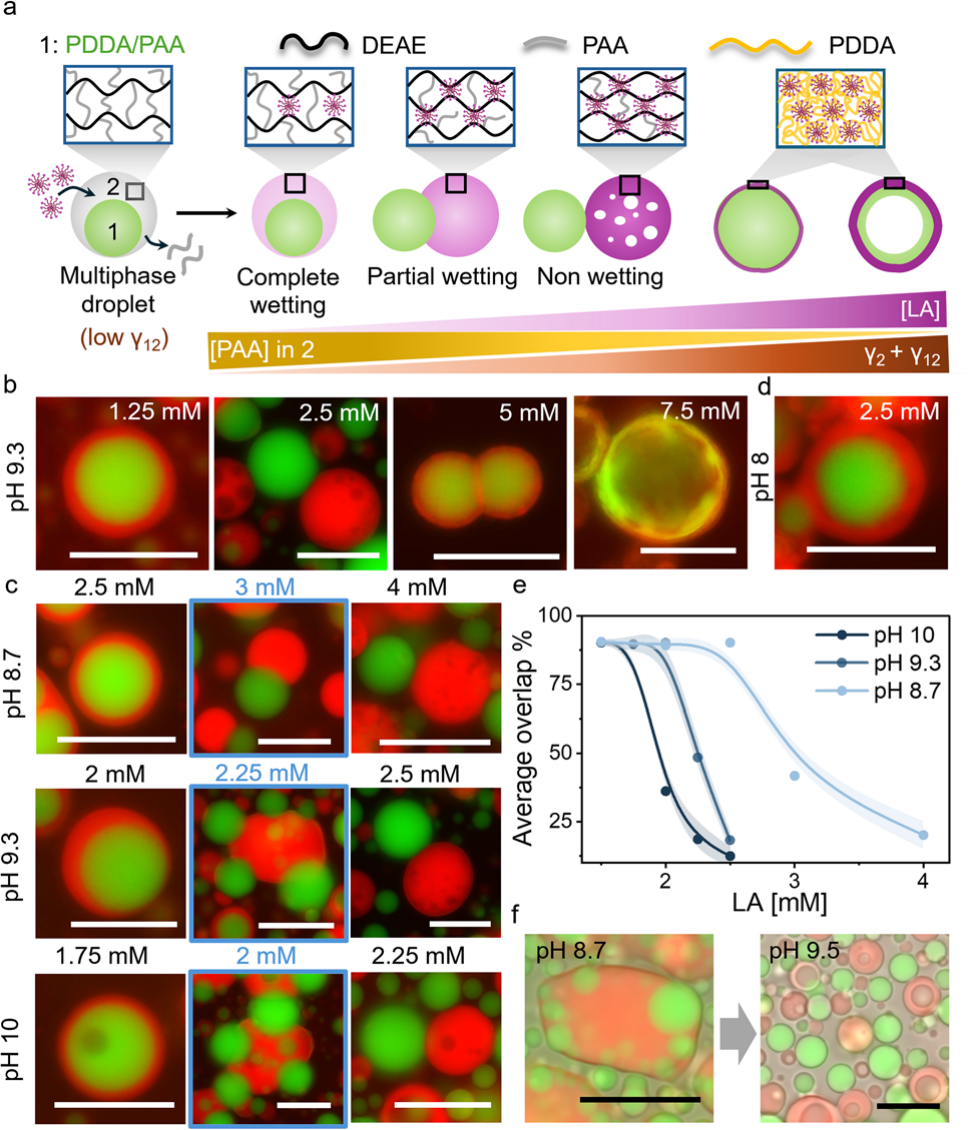
(a) Scheme showing a multiphase coacervate droplet with an inner phase of PDDA/PAA (phase 1, green) and an outer phase of DEAE/PAA (phase 2, grey). Upon addition of LA micelles, they integrate into the outer phase displacing PAA to form a LA/DEAE/PAA phase, leading to an increase in its surface tension (*γ*_2_) and the interfacial tension between the inner and outer phases (*γ*_12_). Above a threshold [LA], the rise in *γ*_12_ and *γ*_2_ leads to partial dewetting, and complete dewetting at higher [LA]. Further addition of LA leads to dissolution of the LA/DEAE/PAA droplet *via* formation of soluble polyelectrolyte complexes and membranization of the PDDA/PAA droplet *via* formation of solid polyelectrolyte complexes of PDDA/LA, which grow at the expense of the inner coacervate phase to transform it into coacervate vesicles at high [LA]. (b) Red and green channel fluorescence microscopy images showing the transition from complete wetting to partial wetting to complete dewetting in multiphase coacervates upon an increase in [LA] at pH 9.3. The red phase is LA/DEAE/PAA, and the green phase is PDDA/PAA. At 5 mM LA, membranized PDDA/PAA droplets are observed, which transform into coacervate vesicles at 7.5 mM LA. (c) The partial wetting condition (blue box) shifts to lower [LA] with an increase in pH. (d) Complete wetting was observed with 2.5 mM LA at pH 8. (e) Plot of average overlap % showing shifting of the dewetting transition (50% overlap) to lower [LA] with an increase in pH from 8.7 to 10. (f) Brightfield/fluorescence microscopy images showing wetting of PDDA/PAA droplets by LA/DEAE/PAA droplets upon mixing at pH 8.7 and dewetting upon increasing the pH to 9.5. All scale bars are 20 µm. Note: Red channel fluorescent label: Nile red (within LA micelles); Green channel fluorescent label: Pyranine (sequestered).

The fatty acid mediated changes in the material properties of DEAE/PAA coacervates were exploited to regulate interfacial tension within multiphase coacervates, where DEAE/PAA constituted the outer phase, and triggered dewetting or droplet division. To this end, we prepared multiphase coacervates, which have an inner phase composed of poly(diallyldimethylammonium) chloride(PDDA)/PAA and an outer phase of DEAE/PAA (Fig. 2a and S9). The addition of LA to these multiphase coacervates at pH 9 led to preferential integration into the outer DEAE/PAA phase, and above a threshold concentration (2.5 mM), the outer phase completely dewetted the inner PDDA/PAA droplet (Fig. 2a, b and S10). The preferential incorporation of LA micelles into the DEAE/PAA phase upon addition of increasing amounts of LA to multiphase coacervates was observed as an increase in red fluorescence intensity (of Nile Red) in the outer phase (Fig. 2a and S11). This change in composition of the outer DEAE/PAA phase *via* gradual incorporation of LA micelles and displacement of PAA, which is a common polyelectrolyte with the PDDA/PAA phase, increased the interfacial tension between the two phases (*γ*_12_) along with the rise in surface tension of the outer LA/DEAE/PAA phase (*γ*_2_) noted before. This rise in interfacial tension upon LA addition at pH 9.3 led to partial dewetting at 2.25 mM LA and complete dewetting and separation of the multiphase droplet into a PDDA/PAA droplet and a LA/DEAE/PAA droplet at 2.5 mM LA (Fig. 2a–c and S10). Notably, the dewetting state was stable in the presence of buffer (pH 9.3) for at least 6 h (Fig. S12), and separately prepared LA/DEAE/PAA droplets and PDDA/PAA (15/45 mM) droplets did not wet each other significantly for at least 2 h when mixed (Fig. S13), suggesting that the dewetting behaviour is occurring at equilibrium. It is also important to note that, in contrast to previous studies where fatty acids have been reported to assemble at the interface to form membranes around coacervate droplets^59,60^ or form lipid rich domains within the coacervate droplets,^61^ in our case we did not observe any interfacial assembly around the multiphase droplets but homogeneous distribution of the LA or OA micelles throughout the DEAE/PAA outer phase. We did however observe interfacial assembly of fatty acids below CMC selectively on positively charged PDDA/PAA droplets (Fig. S14).

To follow the wetting–dewetting processes occurring in large populations of droplets, we used a customised MATLAB script to analyse fluorescence microscopy images (Note S2) and defined a parameter termed as ‘overlap %’, which represented the percentage area of the inner phase overlapping with the outer phase, with 100% and 0% overlap representing complete wetting and complete dewetting conditions, respectively. Upon increasing the concentration of LA added to DEAE/PAA/PDDA multiphase coacervates from 1.75 mM to 2.5 mM at pH 9.3, the average overlap % decreased from 90% to 10%, indicating complete dewetting thoughout the sample at 2.5 mM (Fig. 2e). Interestingly, when the pH was changed to 8 in the presence of 2.5 mM LA, complete wetting was observed (Fig. 2d). The dewetting transition occurred at higher [LA] (4 mM) at pH 8.7 and at lower [LA] (2.25 mM) at pH 10 (Fig. 2c, d and S15). This suggests that at higher pH, the increased charge density of LA micelles enabled better displacement of PAA from the outer phase, thereby allowing pH-based control of multiphase dewetting (Movie S1).

1H NMR studies on the supernatant phase of LA/DEAE/PAA at pH 8.7 and pH 9.5 revealed that all DEAE and most of the LA were present in the coacervate phase at lower pH, and at higher pH the concentration of all three components (DEAE, LA and PAA) increased in the supernatant. This suggested that at pH 8.7, the protonated form of LA is present within the coacervate phase, and at pH 9.5, the increase in concentration of the deprotonated form of LA leads to formation of soluble polyelectrolyte complexes with DEAE, releasing PAA also into the supernatant (Note S1). Furthermore, when LA/DEAE/PAA (5/2.4/17.3 mM) coacervate droplets were mixed with PDDA/PAA (15/45 mM) droplets at pH 8.7, we observed complete wetting interactions between the two droplets to form multiphase droplets, and upon raising the pH by addition of carbonate buffer (pH 9.8), the outer LA/DEAE/PAA phase vacuolized, rapidly dewetting the PDDA/PAA droplets (Fig. 2f, Note S1 and Movie S1). The observed vacuolization in the outer phase was consistent with the formation of soluble polyelectrolyte complexes upon an increase in pH, as noted in the 1H NMR studies. The above results indicate that the outer phase composition can be regulated by changes in pH to control wetting interactions. Notably, similar dewetting behaviour in multiphase coacervates was also observed upon addition of oleic acid (OA, 1.75 mM) at pH 10, which is above the p*K*_a_ of OA (p*K*_a_ = 9.85 in its self-assembled state; Fig. S16). This suggests that the regulation of multiphase wetting interactions could be observed with other fatty acids as well.

Further increase in fatty acid (FA; LA or OA) concentration beyond the dewetting transition caused vacuolization of the dewetted FA/DEAE/PAA droplet and its gradual dissolution due to formation of soluble polyelectrolyte–fatty acid complexes (Fig. 2a, b, S10, S15 and S16). At 4 mM LA or 3.5 mM OA, FA started to interact with PDDA/PAA droplets, forming a PDDA/FA polyelectrolyte complex membrane at the surface by displacing PAA (Fig. 2a, b, S10 and S15–S17). Further increase to 7.5 mM LA or OA led to growth of the PDDA/FA membrane at the expense of the PDDA/PAA coacervate, which mostly disappeared, forming two layered coacervate vesicles with a PDDA/FA membrane and an underlying PDDA/PAA shell (Fig. 2a and c). However, it is important to note that LA has preferential interaction with DEAE over PDDA, and its enrichment in the DEAE/PAA outer phase is not a kinetic state. This was shown by experiments where LA was added to PDDA/PAA coacervate droplets leading to the formation of PDDA/LA aggregates that disappeared upon addition of DEAE, which formed an outer DEAE/PAA phase and subsequently sequestered LA (Fig. S18). Overall, these results suggest that fatty acids can regulate the material properties of phases selectively to control multiphase wetting interactions and also induce morphological transformations such as selective phase dissolution, membranization and coacervate vesicle formation.

### pH-responsive switching of multiphase wetting interactions

**Fig. 3:**
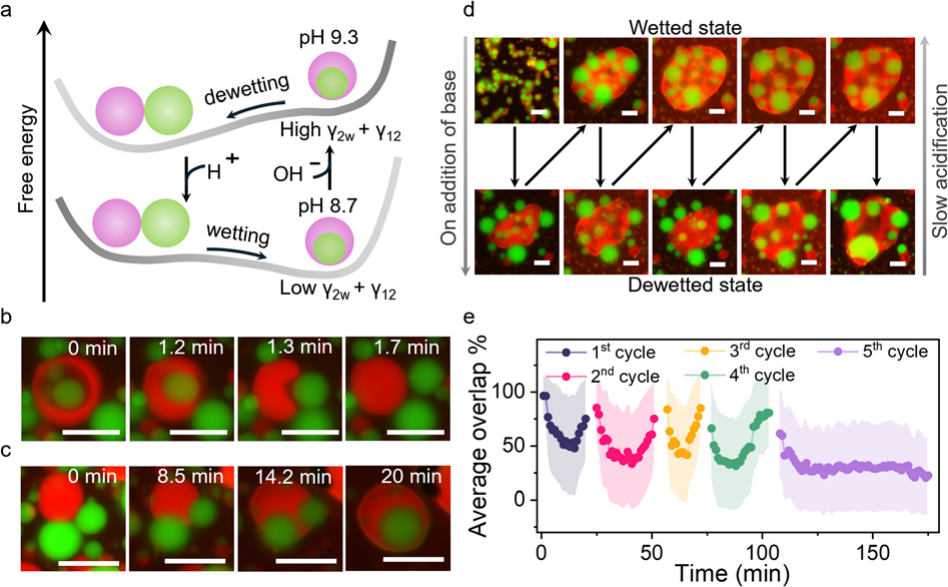
(a) Scheme showing the change in the free energy surface of the LA/DEAE/PAA/PDDA multiphase droplet, leading to spontaneous dewetting and wetting upon changing the pH to 9.3 and pH 8.3, respectively. Fluorescence microscopy images showing (b) dewetting of the PDDA/PAA (green) droplet by the outer LA/DEAE/PAA (red) droplet upon an increase in pH and (c) re-wetting upon a decrease in pH. (d) Red and green channel fluorescence microscopy images showing multiple cycles of wetting and dewetting of LA/DEAE/PAA/PDDA multiphase coacervate droplets (2.5 mM LA) upon local addition of 4 µL of buffer (carbonate buffer, pH 10) adjacent to the viewing area into multiphase coacervate droplets at pH 8.3, and the corresponding average overlap % plots are shown in (e). All scale bars are 20 µm. Note: Red channel fluorescent label: Nile red (within LA micelles); Green channel fluorescent label: Pyranine (sequestered).

After studying the interactions of multiphase coacervates with unsaturated fatty acids (LA and OA) with respect to changes in concentration and pH, we attempted to reversibly reconfigure the multiphase droplets between their core–shell architecture and complete non wetting state by locally regulating the pH (Fig. 3a). The addition of an aliquot of carbonate buffer (pH 10, 4 µL) to multiphase coacervates containing 2.5 mM of FA at pH 8.3 (for LA) and pH 9.3 (for OA) raised the pH locally above the FA's p*K*_a_ and triggered the complete dewetting of the two phases due to increase in *γ*_12_ and *γ*_2_ (Fig. 3a, S19 and S21). The lowering of pH due to atmospheric CO_2_ dissolution in the sample led to the complete engulfment of the PDDA/PAA droplets by the FA/DEAE/PAA phase due to decrease in *γ*_2_ and *γ*_12_ (Fig. 3a, S19 and S21). The process of dewetting was relatively quick with the rise in pH due to buffer being immediate and took only 1-2 min in the case of both LA and OA (Fig. 3b, Movies S2 and S3). In contrast, the wetting of the droplets was relatively slow taking up to 20 and 10 min, in the case of LA and OA, respectively (Fig. 3c, S21, S22, Movies S2 and S3). The slower re-wetting in case of LA was due to the slower acidification of the sample at a less basic pH, *i.e.* pH 9 rather than pH 10 as in the case of OA.

Four cycles of reconfiguration of multiphase droplet populations could be carried out by repeated addition of aliquots of buffer before the accumulation of buffer hindered the process (Fig. 3d, S19, S20 and Movies S4–S6). The average overlap % plots determined from processing of fluorescence microscopy images of the area adjacent to the point of addition of the buffer showed four complete cycles of dewetting and re-wetting, with the fifth cycle showing a decrease in overlap % (dewetting) but no recovery was noted (Fig. 3e). The above results show that by phase selective incorporation of fatty acids within the outer phase of the multiphase droplet, we have been able to achieve pH-responsive switching of multiphase wetting interactions.

### Chemical signalling mediated reconfiguration of multiphase coacervate droplets

**Fig. 4:**
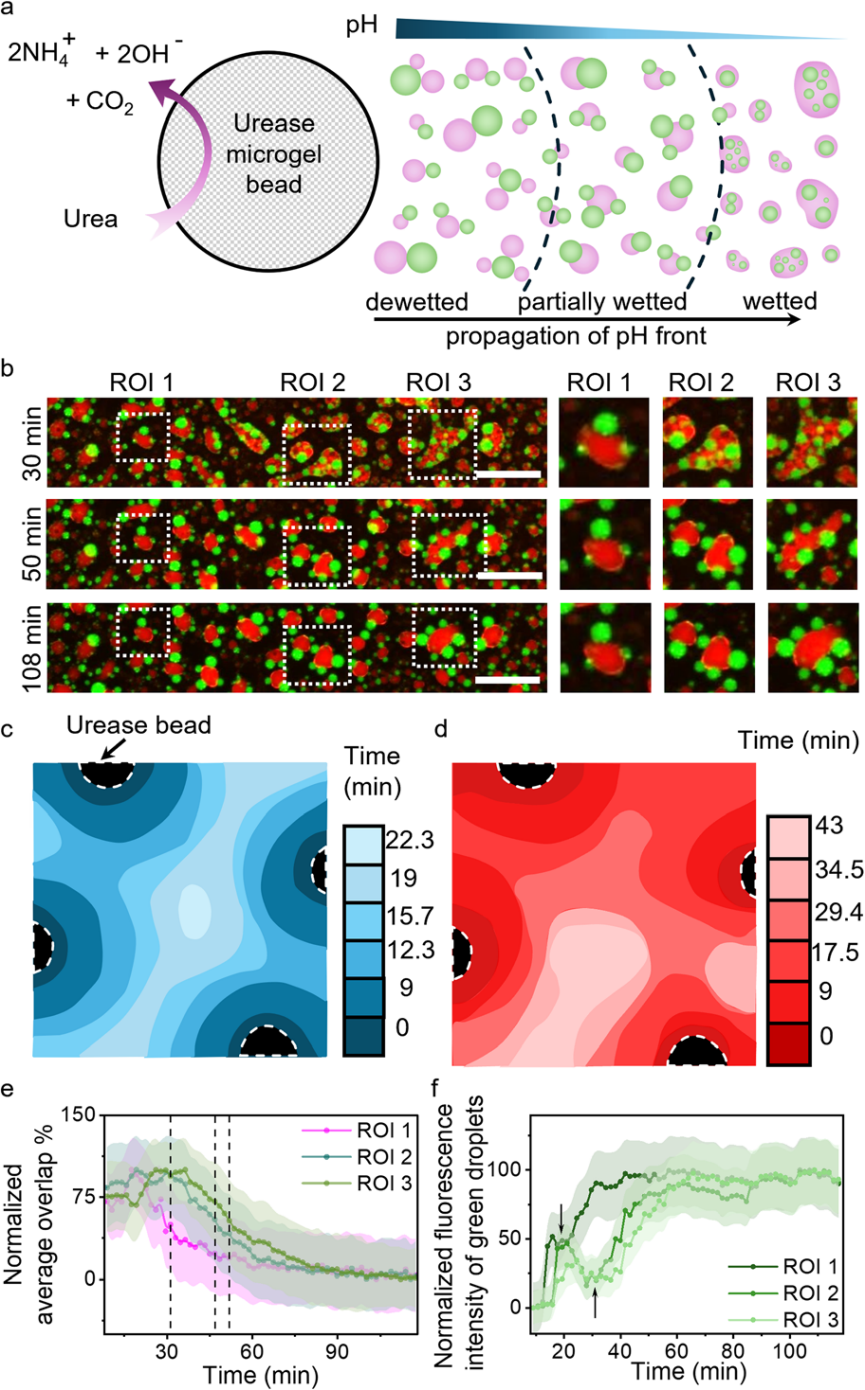
(a) Scheme showing the outward propagation of a basic pH front from a urease microgel triggering a reconfiguration front within multiphase coacervate droplets. (b) Time sequence of red and green channel fluorescence microscopy images showing a dewetting front triggering dewetting in multiphase coacervate droplet population in ROI 1, ROI 2, and ROI 3 regions. Note: Red channel fluorescent label: Nile red (within LA micelles); Green channel fluorescent label: Pyranine (sequestered). The basic pH front first triggered vacuolization in the outer phase, which was used to identify the propagation of the coacervate response front shown in (c) and subsequently caused dewetting identified as the reconfiguration front shown in (d). (e) Plot showing a decrease in overlap % of multiphase coacervate droplets in the different ROIs shown in (b). A faster decrease is noted in ROI 1 followed by ROI 2 and ROI 3, and dashed lines identify the dewetting transition (50% overlap) for ROIs 1, 2 and 3 at 31, 47 and 52 min, respectively. The initial rise in overlap % is an artifact due to vacuolization of the outer phase obscuring the detection of the wetting state. (f) Plot of normalized average fluorescence intensity of PDDA/PAA droplets for the different ROIs, showing a faster rise in ROI 1 followed by ROI 2 and ROI 3. The dip (black arrows) in fluorescence intensity observed is an artifact due to the vacuolization of the outer phase. Scale bar is 200 µm.

To showcase the potential of this work in terms of synthetic cells, we employed urease containing Ba-alginate microgels to secrete pH-based chemical signals (ammonia) and set up a cascade of reconfiguration within multiphase coacervate droplet populations (Fig. 4a). Four urease microgels were deposited at the edges of the viewing area, separated by distances of *ca.* 3.1 mm, containing multiphase droplets with 2.5 mM LA at pH 8.3 in the presence of urea (4 mM) (Fig. S23). Urease microgels (∼650 µm) converted urea into ammonia and carbon dioxide, raising the pH of the local environment and setting up an outward propagating basic pH front, which was followed *via* the increase in green fluorescence of pH-sensitive dye, Pyranine, within PDDA/PAA droplets (Fig. S24). For illustrating the processes occurring in the viewing area, 3 ROIs were identified within a small section of it where the average green fluorescence of the PDDA/PAA droplets was tracked to reveal the movement of the basic pH front from left to right (Fig. 4b and f). The pH fronts from the four beads converged within the viewing area, causing a reconfiguration cascade in the multiphase droplet population (Fig. 4b–d and S23). The propagating basic pH front first triggered vacuolization in the outer DEAE/PAA phase due to increased charge-based interactions with the LA micelles, which can be followed *via* analysis of brightfield microscopy images to reveal the speed of the coacervate response (vacuolization) front to be *ca.* 160 µm min^−1^ (Fig. S23 and Movie S7). The coacervate response fronts coming from the four urease beads converged at the center within 23 min (Fig. 4c). The vacuolization of the outer phase was followed by the dewetting of the multiphase droplet, which was tracked *via* analysis of overlap % using MATLAB (Note S2) for processing of red/green channel fluorescence microscopy images to follow the front of reconfiguration propagating through the multiphase droplet population (Fig. 4b, d, e, Movie S8 and Fig S25, S26). The reconfiguration fronts from the four beads converged within the viewing area at *ca.* 43 min, indicating that the dewetting process takes *ca.* 20 min to complete from the time of arrival of the basic pH front. The % overlap plots for the 3 ROIs showed that dewetting transition, identified as 50% overlap, occurred at *t* = 31 min, 46 min and 52 min for ROIs 1, 2 and 3, respectively, illustrating the spatiotemporal control over wetting interactions in multiphase droplets *via* chemical signaling (Fig. 4e). The above results show that colloids secreting pH-based chemical signals can be used to spatiotemporally reconfigure the structure of multiphase droplet populations in the local environment.

## Conclusion

To conclude, we have shown that enrichment of unsaturated fatty acids within DEAE/PAA coacervates drastically changed their material properties such as viscosity and surface tension. By constituting a multiphase droplet with DEAE/PAA as the outer phase, the selective enrichment of fatty acids within the outer phase was shown to modulate material properties of the outer phase and control multiphase wetting in a concentration dependent manner. The pH-responsive dynamic regulation of multiphase wetting interactions was achieved by changes in pH near the p*K*_a_ of the fatty acid and chemical signaling mediated spatiotemporal reconfiguration of multiphase droplets was illustrated. To the best of our knowledge, this work represents the first report of small molecule-based regulation of multiphase wetting interactions, and the complete dewetting observed in our case is a manifestation of drastic change in material properties of the coacervate mediated by small molecules. Our results would find applications in the field of synthetic cells where the transduction of chemical signals into structural response would diversify the existing toolbox for implementation of complex regulatory networks necessary for design of collective behaviour in synthetic cell communities. Regarding condensates, our results indicate that lipids enriched within condensates could potentially be playing a key role in regulating their morphology and function.^57^

## Author contributions

P. S. – methodology, data curation, formal analysis, investigation, validation, visualization and writing. P. S. P – software (MATLAB code for image analysis). B. V. V. S. P. K. – conceptualization, funding acquisition, project administration, resources, supervision and writing.

## Conflicts of interest

There are no conflicts to declare.

## Supplementary Material

SC-017-D5SC07783D-s001

SC-017-D5SC07783D-s002

SC-017-D5SC07783D-s003

SC-017-D5SC07783D-s004

SC-017-D5SC07783D-s005

SC-017-D5SC07783D-s006

SC-017-D5SC07783D-s007

SC-017-D5SC07783D-s008

SC-017-D5SC07783D-s009

## Data Availability

Raw data that support the findings of this study are available from the corresponding author upon reasonable request. The authors confirm that the data supporting the findings of this work are available within the article and its supplementary information (SI). Supplementary information: methods, Notes S1 and S2, Movies S1–S8 and Fig. S1–S26. See DOI: https://doi.org/10.1039/d5sc07783d.
