# Supplementary material for: pH-responsive regulation of multiphase coacervate wetting via phase selective enrichment of fatty acids
Source: Chem Sci. 2025 Dec 11;17(6):3285–92. doi: 10.1039/d5sc07783d (PMC12716314; doi:10.1039/d5sc07783d)
Supplement: SC-017-D5SC07783D-s009 [file SC-017-D5SC07783D-s009.pdf]

Supplementary Information (SI) for Chemical Science.
This journal is © The Royal Society of Chemistry 2025

pH-responsive regulation of multiphase coacervate wetting via phase selective enrichment of fatty acids

Preeti Sharma,[a] Pankaj Singh Patwal[a] and B.V.V.S. Pavan Kumar[a]*

[a] Dynamic Colloidal Systems Laboratory, Department of Chemistry, Indian Institute of Technology Roorkee, Roorkee-247667, India
E-mail: pavan.bosukonda@cy.iitr.ac.in

Contents

Materials and Methods 2
- Materials 2
- Preparation of coacervate microdroplets 2
- Turbidity studies 2
- pKa study of linolenic acid 2
- Microscopy imaging 3
- Functionalisation of glass coverslips 3
- Fluorescent labelling of DEAE 3
- Protocol for NMR-based coacervate component analysis 3
- Microdroplet fusion dynamics 3
- Microrheology studies 4
- Fluorescence recovery after photobleaching studies 5
- Protocol for water content estimation in different coacervate systems 5
- SAXS study of different coacervate systems 6
- Unsaturated fatty acid mediated dewetting of multiphase microdroplets 6
- Cycling of dewetting/wetting of multiphase coacervates mediated by unsaturated fatty acids 6
- Preparation of urease containing gel beads 6
- Urease gel bead mediated propagation of dewetting front through a population of multiphase coacervate droplets 6

Note S1 7

Note S2 12

Supplementary videos 14

Supplementary Figures 15

Materials and Methods

Materials

All materials were used as received. Poly(diallyldimethylammonium) chloride (PDDA, M_w - (200-350) kDa, Polyacrylic acid sodium salt (PAA, M_w - 5100 by GPC), Diethylaminoethyl-dextran hydrochloride (DEAE) (M_w - 500 kDa), Alginic acid sodium salt from brown algae, Chitosan (medium molecular weight), oleic acid (technical grade, 90%), α-linolenic acid (≥ 99%), Urease from jack bean (50,000-100,000 U/g), urea, glucose, pyranine (HPTS) and Rhodamine isothiocyanate (RITC), were purchased from Sigma, dibutyltin dilaurate from TCI, 1 µm polybead carboxylate microspheres was purchased from Polysciences, N-(triethoxysilylpropyl)-o-polyethylene oxide urethane purchased from Gelest, dialysis membrane (MWCO ≈ 12-14 kDa) obtained from Himedia, Barium chloride, nile red, toluene, ethanol were purchased from SRL. All the stock solutions were prepared using double-deionized water.

Preparation of coacervate microdroplets

In this study, three different single phase coacervate systems, viz, PDDA/PAA, DEAE/PAA, and DEAE/LA, and a multiphase coacervate system, viz, DEAE/PAA/PDDA, were investigated. Single phase coacervate samples were prepared by mixing oppositely charged polyelectrolytes at various concentrations. Typically, multiphase coacervate microdroplets were formed by initially preparing a PDDA/PAA coacervate (10 mM/40 mM, i.e. 10 mM PDDA, 40 mM PAA) with excess PAA to form a negatively charged coacervate, then DEAE (2.21 mM) was added to form multiphase coacervate. To visualize the different phases of the DEAE/PAA/PDDA multiphase coacervate in fluorescence microscopy, pyranine and RITC-DEAE were used. Pyranine showed a higher partition coefficient for PDDA/PAA phase than for DEAE/PAA phase which was used to label the PDDA/PAA droplets green. To label the DEAE/PAA phase we used RITC-DEAE in initial experiments but later the red channel label was used to track the presence of unsaturated fatty acids (Linolenic acid (LA) and Oleic acid (OA)) in the coacervate phases. The coacervate microdroplets, both single and multiphase, were prepared at pH 8, unless specified otherwise. The pH of the polyelectrolyte solutions were adjusted using 1 M HCl or 1 M NaOH before use in preparation of coacervates.

Turbidity studies

The turbidity measurements on coacervate dispersions were used to investigate: the pH range of existence for different coacervate systems by adjusting the pH using 1 M HCl and 1 M NaOH; the monomer ratio of polyanion/polycation for the formation of different coacervates; and critical salt concentration (CSC) for the dissolution of different coacervate systems. Turbidity measurements were conducted using a Shimadzu UV-2600 UV/VIS spectrometer at room temperature. The absorbance of the coacervate samples was measured at specific wavelengths for different coacervate systems: 600 nm for PDDA/PAA, and 620 nm for DEAE/PAA and DEAE/LA respectively. The measurements were performed using polystyrene cuvettes with a path length of 1 cm.

pKa study of linolenic acid

The pK_a value of linolenic acid was determined using a pH titration. The linolenic acid solutions (0.5 mM and 1.5 mM, 40 mL) are set to a high starting pH using 0.047 M NaOH (3 mL). The pH titration was carried out by adding aliquots of 40 µL (0.145 N HCl) with continuous measurement of pH. The pH titration data were used to plot the dpH/dV curve to identify the equivalence points, and the pKa values were obtained from the half-equivalence point of each COOH group. The pH titration data revealed pK_a values of ~6.5 and 7.8 for 0.5 mM LA and 1.5 mM LA, respectively.

Microscopy imaging
Olympus IX83 inverted fluorescent microscope was used for optical and fluorescence microscopy. The specifications of the fluorescence filter cubes used for different channels were the following: Green Channel (for FITC and Pyranine) - λ_{ex} : 470-495 nm, λ_{em} : 510-550 nm; Red Channel (for RITC) - λ_{ex} : 540-550 nm, λ_{em} : 575-625 nm.

DEAE/PAA/PDDA multiphase microdroplets were imaged using RITC-DEAE (30 µM) to follow the DEAE/PAA phase and pyranine (9 µM) to follow the PDDA/PAA phase.

In all experiments involving unsaturated fatty acids, to follow the sequestration/incorporation of unsaturated fatty acids into the DEAE/PAA phase we used Nile Red (9 µM, red channel) and to follow the PDDA/PAA droplets, we used Pyranine (9 µM, green channel), which preferentially sequestered in the PDDA/PAA droplets.

Functionalisation of glass coverslips
To avoid the wetting behaviour of coacervate droplets, coverslips were functionalized using PEG-silane as previously reported[1]. Briefly, the procedure used was as follows: first, the coverslips were rinsed with ethanol followed by water and blow dried with air. This was followed by piranha treatment for 1 h and then 1 h treatment in base bath, then washed with ethanol and water. The coverslips were incubated for 4 h in a 0.3 wt% solution of N-(triethoxysilylpropyl)-o-polyethylene oxide urethane in toluene. The functionalized coverslips were thoroughly washed with toluene, ethanol, and water before drying overnight at 70 °C. For microscopy, the silane-coated coverslips were glued to the bottom of pierced plastic petri dishes. Similar procedure was used for silanizing glass capillaries for other studies.

Fluorescent labelling of DEAE
We employed a previously reported[2] protocol for Rhodamine-B labelling of DEAE. In brief, 50 mg of DEAE was dissolved in 5 mL of anhydrous DMSO at 70 °C, followed by addition of 50 mg sodium hydrogen carbonate, 20 µL of dibutyltin dilaurate, and 20 mg of RITC. The mixture was then stirred at 70 °C in the dark for 16 h and then dialyzed the reaction mixture against double-deionized water for 3 days to isolate the tagged polymer.

Protocol for NMR-based coacervate component analysis
To quantify the content of DEAE, PAA, and LA in the coacervate phase, the 1H NMR of supernatant phase was recorded, NMR peaks corresponding to characteristic protons were identified and their peak areas were analysed. All DEAE, PAA, and LA solutions were prepared in D_2O and adjusted to pH 9 using DCl and NaOD. DEAE/PAA coacervates (2.4 mM/ 17.3 mM) were prepared and different amounts of LA (0, 2, 5, 6, 7 and 8 mM) were added to them. The total volume of coacervate dispersion prepared was 1 mL. These coacervate systems were centrifuged and the supernatant (0.7 mL) was collected. 0.1 mL of p-toluene sulfonic acid (pTSA, 1.7 mM) was added as an internal reference and 1H NMR spectra was recorded. It is important to note that in case of PAA, the supernatant was acidified (to get separate peaks for PAA, as in basic medium, peaks are overlapping with LA and DEAE) by adding 10-20 µL of DCl, then the solution was centrifuged, and 0.7 mL of solution was collected for 1H NMR analysis. Peak areas under the respective different NMR peaks were analyzed for the quantification of the DEAE, PAA, and LA. The peak area was corrected (15%) for the dilution of the sample due to internal reference (pTSA) addition and in the NMR peak area table (Note S1) corrected peak area was mentioned.

Microdroplet fusion dynamics
The microdroplet fusion studies[3] of PDDA/PAA (30 mM/40 mM), DEAE/PAA (5.5 mM/34 mM), LA/DEAE/PAA (2 mM/2.4 mM/17.3 mM), and LA/DEAE/PAA (5 mM/2.4 mM/17.3 mM) coacervates were carried out in PEG-silane functionalized petri dishes. To follow the fusion of the coacervate microdroplets, brightfield microscopy images were captured at every 0.1 s interval. For each image, the coacervate aspect ratio AR(t) = L/l, where 'L' and 'l' are the lengths of the long and short axis of

the coacervate droplets, respectively, was measured using the ImageJ software. The shape relaxation time, τ, was obtained by fitting to an exponential fit as, $AR(t) = a + b\cdot\exp(-t/\tau)$. The characteristic length 'l' is the coacervate radius after fusion, and the relaxation time is directly proportional to the characteristic length according to the relation, $\tau = (\eta/\gamma)\cdot l$, and the inverse capillary velocity is the ratio of viscosity and surface tension of the coacervate. To calculate the surface tension of the coacervate droplets, we carried out microrheology studies to determine 'η' using Stokes-Einstein equation and used the value of η/γ from fusion dynamics study to determine γ.

Microrheology studies

Microrheology of coacervate droplets [PDDA/PAA (30 mM/40 mM), DEAE/PAA (5.5 mM/34 mM), LA/DEAE/PAA (2 mM/2.4 mM/17.3 mM), LA/DEAE/PAA (5 mM/2.4 mM/17.3 mM), and LA/DEAE (5 mM/2.4 mM)] were studied using the previously described method[3,4]. Briefly, 0.5 µm Polybead® carboxylate microspheres (20 µL), were dispersed into phosphate buffer solution (pH 8, 2 mL) and then 20 µL of it was added to the coacervate solution (pH 8) and centrifuged to isolate the coacervate phases with microspheres. The coacervate phase was transferred to a 0.4 mm glass capillary which was later sealed from both ends using nail paint. The movement of fluorescent microparticles (avoided beads at the bottom of the glass capillary) in coacervate phase were tracked using a fluorescence microscope for 1 h at 1.5 s intervals. After that images were analyzed using ImageJ software and the x and y coordinates of particles (number of particles tracked ≥ 17) were obtained over time using 'trackmate' plugin. MSD of the microparticles was calculated using MATLAB and fitted to an equation $MSD(\tau) = 4D\tau^{\alpha}$, where α is a diffusive exponent ($\alpha = 1$, for Brownian motion of the beads). The viscosity of coacervate droplets was calculated using the Stokes-Einstein equation, $D = k_BT/6\pi\eta r$, where k_B is Boltzmann constant, T is temperature, η is the viscosity of the coacervates and r is the radius of the fluorescent beads.

MSD was calculated from time and ensemble averages for all trajectories:

$$MSD(\tau)=\langle(x(\tau+t) - x(t))^2\rangle+\langle(y(\tau+t) - y(t))^2\rangle, \text{ where } \tau \text{ is the lag time}$$

The diffusion coefficients (D) of 1 µm microspheres in PDDA/PAA, DEAE/PAA, LA (2 mM)/DEAE/PAA, LA (5 mM)/DEAE/PAA, and LA/DEAE were found to be 41.5 x 10^{-5} µm^2/s, 103 x 10^{-5} µm^2/s, 33.5 x 10^{-5} µm^2/s, 2.6 x 10^{-5} µm^2/s, and 0.49 x 10^{-5} µm^2/s, corresponding to viscosities of 2.1 Pa.s, 0.8 Pa.s, 1.30 Pa.s, 16.79 Pa.s, and 88.19 Pa.s respectively.

From fusion dynamics studies we obtained inverse capillary velocity values (η/γ) of 0.062, 0.032, 0.063, and 0.578 for PDDA/PAA, DEAE/PAA, LA (2 mM)/DEAE/PAA, and LA (5 mM)/DEAE/PAA respectively. This gave the following values for surface tension of the different coacervate systems as:

$\gamma_{\text{PDDA/PAA}}$ = 33.9 µN/m, $\gamma_{\text{DEAE/PAA}}$ = 25.0 µN/m, $\gamma_{\text{LA (2 mM)/DEAE/PAA}}$ = 20.6 µN/m, and $\gamma_{\text{LA (5 mM)/DEAE/PAA}}$ = 29.0 µN/m, respectively.

The MATLAB code used for the MSD analysis of the microparticles is as follows:

```
a

for j =1:length(a)-1

for i=1:length(a)-j

msd =(a(i+j,1)-a(i,1)).^2+(a(i+j,2)-a(i,2)).^2

v(j,i)=msd

end
```

k=sum(v,2)

end

for g=1:length(a)-1

r(g,1)=k(g,1)/(length(a)-g)

end

where “a” includes x -y coordinates,

“r” includes mean MSD of each lag time.

Fluorescence recovery after photobleaching studies

FRAP data of four different coacervate systems DEAE/PAA (2.4 mM/ 17.3 mM), LA/DEAE/PAA (2 mM/ 2.4 mM/ 17.3 mM), LA/DEAE/PAA (5 mM/ 2.4 mM/ 17.3 mM), and LA/DEAE (5 mM/ 2.4 mM) were studied using the previously described method[4]. The sample preparation was done as follows: RITC-c-BSA was added to the four different coacervate systems. The droplets were allowed to settle onto the glass coverslip for about five minutes before recording the data. Four different coacervate systems were studied using FRAP on an upright Leica STELLARIS 5 confocal microscope. A circular spot with a diameter of 0.5 μm at the centre of a droplet was selected for photobleaching, and four distinct coacervate droplets from the same sample were used to record measurements. Using ImageJ, the fluorescence after photobleaching was measured to detect fluorescence recovery. Double normalization was used to standardize the data.

$$FN(t) = [P(t) - B(t)].[R(0) - B(0)]/R(t) - B(t)].[P(0) - B(0)]$$

where R(t) is the reference fluorescence intensity of the droplets encircling the bleached area, B(t) is the intensity of an arbitrary chosen background region, P(t) is the average fluorescence intensity within the photobleached area, and $F_N(t)$ is the normalized fluorescence intensity. Data was fit to a single exponential function: FN(t) = A1*exp(x/τ) + y_0, where τ is the time constant for fluorescence recovery respectively. The recovery half-time $τ_{1/2}$ was calculated as: $τ_{1/2} = τ_f \ln(2)$,

Name	τ (s)	$τ_{1/2}$ (s)
DEAE/PAA	65.7	45.2
LA/DEAE/PAA (2 mM)	232	160.8
LA/DEAE/PAA (5 mM)	320	221.8
LA/DEAE (5 mM)	527	365.3

Table S1: Table showing recovery half-time $τ_{1/2}$ for different coacervate systems.

Protocol for water content estimation in different coacervate systems

Different coacervate systems were centrifuged: DEAE/PAA (2.4 mM/17.3 mM), LA/DEAE/PAA (2 mM/2.4 mM/17.3 mM), and LA/DEAE/PAA (5 mM/2.4 mM/17.3 mM). Later, the supernatant phase was removed to obtain the dense coacervate phase, and the different dense coacervate phases were weighed before being freeze-dried. After that, the dense coacervate phase was freeze-dried for over 24 hours to remove water, and then the weight of the freeze-dried coacervate phase was measured. Then, % water was calculated with the formula.

$$\%water = 100\,X\,(weight\ before\ drying - weight\ after\ drying)/weight\ before\ drying$$

Coacervate system	% Water
**D/P**	86 %
**L2/D/P**	81 %
**L5/D/P**	70 %

Table S2: Weight percentage of water in different coacervate phases estimated by freeze drying of the centrifuged bulk coacervate phase.

SAXS study of different coacervate systems

SAXS studies were carried out on different coacervate systems: DEAE/PAA (2.4 mM/17.3 mM), LA/DEAE/PAA (2 mM/2.4 mM/17.3 mM), LA/DEAE/PAA (5 mM/2.4 mM/17.3 mM), and LA/DEAE (5 mM/2.4 mM). First, all the different coacervate solutions were centrifuged, and the dense coacervate phase was collected after removing the supernatant. Later, the dense coacervate phase was loaded into PasteCell for a viscous solution to collect SAXS data. The SAXS data were collected using an Anton Paar SAXS 2.0 instrument.

Unsaturated fatty acid mediated dewetting of multiphase microdroplets

The DEAE/PAA/PDDA multiphase coacervates (2.21 mM/ 40 mM/ 10 mM) were exposed to different concentration of fatty acids (1 - 5 mM oleic acid (OA), or 1.25 - 7.5 mM linolenic acid (LA)) to study the dewetting response of the coacervate system while maintaining the pH of the solution at different values (pH 10, 9.3 and 8.7 for LA; pH 10 for OA) around the pK_a of the unsaturated fatty acid : pK_a of OA = 9.85 [5]; pK_a of LA = 8.28 [5]. The pH of the solution was adjusted to different values using 1 M HCl and 1 M NaOH.

Cycling of dewetting/wetting of multiphase coacervates mediated by unsaturated fatty acids

The multiphase coacervate droplets (DEAE/PAA/PDDA (2.21 mM/40 mM/10 mM)) exposed to unsaturated fatty acids (2.5 mM OA or LA) at pH 9 (for OA) or at pH 8.3 (for LA), were cycled between their wetted and dewetted states by addition of aliquots (4 µL) of carbonate buffer (0.05 M, pH 10). The addition of aliquots of buffer to the sample, raised the pH locally as the buffer diffused into the surrounding medium triggering the dewetting of the multiphase coacervate microdroplets. The pH again dropped back passively via dissolution of CO_2 into the sample from the atmosphere causing wetting interactions and formation of multiphase droplets. Three to four consecutive cycles of dewetting/wetting were carried out, after which the gradual accumulation of buffer in the environment did not allow the cycling of pH. The average overlap percentage (0 to 100%) was analysed using MATLAB code specifically written for this purpose (Note S2), which was also used to color-code the PDDA/PAA droplets based on their overlap %.

Preparation of urease containing gel beads

A previously reported protocol[6] was adapted for the preparation of Barium alginate gel beads containing urease/glucose oxidase as follows: 2 wt% Na-alginate solution containing enzymes (10 mg/mL urease) was extruded into microdroplets using a coaxial airjet (1 L/min) which were collected in a 5 mL glass vial containing 50 mM $BaCl_2$ solution with 0.1% TWEEN 20 and 0.8 wt% chitosan. The entry of the microdroplets into the chitosan solution lead to formation of chitosan/alginate microcapsules whose interior was simultaneously gelled over a period of 20 minutes in the presence of barium chloride (50 mM) in the chitosan solution. The prepared barium alginate gel beads were washed several times with water and finally stored in a 10 mM $BaCl_2$ solution for further use.

Urease gel bead mediated propagation of dewetting front through a population of multiphase coacervate droplets

To start with, a dispersion of multiphase coacervate droplets (DEAE-Dex/PAA/PDDA (2.21-40-10 mM)) with linolenic acid (2.5 mM) and urea (4 mM) at pH 8.3 were added to a glass bottom petri dish

and allowed to settle for 15 min. Then, one or more barium alginate beads containing urease (10 mg/mL) were added, and the petri dish was sealed with a coverslip on top. The urease gel bead mediated reconfiguration of the multiphase droplets in the local microenvironment was followed using fluorescence and brightfield microscopy for about 2 h. The fluorescence microscopy images were processed with a MATLAB script (Note S2) to analyse for variation in overlap %. Different ROI's at increasing distance from the beads were analysed to follow the propagation of a basic pH front triggering reconfiguration of multiphase droplets.

Note S1

NMR-based component-wise analysis of dilute phase

To quantify the components PAA, DEAE, and fatty acid (LA) in the LA/DEAE/PAA coacervate phase with increase in [LA] (2, 5, 6, 7 and 8 mM), the supernatant phase was analyzed via 1H NMR. The following protons corresponding to different components were chosen for 1H NMR analysis: PAA (3H)[7]: —C**H**(COOH)—C$\mathbf{H_2}$— ; DEAE (6H)[8]: —N($CH_2C\mathbf{H_3})_2$; LA (3H)[9]: —C$\mathbf{H_3}$. The 1H NMR analysis of the supernatant phase for the 3-component analysis was done in two steps. The isolated supernatant phase was directly mixed with p-Toluenesulfonic acid (pTSA, internal reference) and 1H NMR was recorded for analysis of peak areas of DEAE and LA. In the case of PAA, the supernatant phase was acidified with DCl to avoid overlap of the peaks with those corresponding to LA and DEAE and then pTSA was added before recording 1H NMR.

The addition of increasing amounts of LA, led to release of PAA into the supernatant and depletion of DEAE from the supernatant, suggesting that incorporation of LA into the DEAE/PAA coacervate phase triggered displacement of PAA and sequestration of DEAE from supernatant due to stronger interactions with LA. With an increase in [LA], the area under the PAA peaks increased (green shaded regions in **Figures A and B**), showing the release of PAA in the supernatant. While DEAE (light red shaded regions in **Figures A and B**) showed a decrease in the area under the peaks. While the area under the LA peaks (grey shaded regions in **Figures A and B**) was also increasing due to an increase in LA concentration added.

One set of NMR data is shown in Figures A and B below. Two data sets were recorded. A table summarising the area under the peaks corresponding to selected protons of DEAE, PAA, and LA is shown below.

Table of peak area of supernatant of different coacervate systems:

Name	DEAE peak area	LA peak area	PAA peak area (after acidification)
DEAE/PAA	5.8	0	4.9
DEAE/PAA/LA (2 mM)	4.1	0.5	6.9
DEAE/PAA/LA (5 mM)	2.3	0.8	8.1
DEAE/PAA/LA (6 mM)	18.5	12.2	17.6
DEAE/PAA/LA (7 mM)	18.9	14.7	17.6
DEAE/PAA/LA (8 mM)	18.8	16.2	17.5

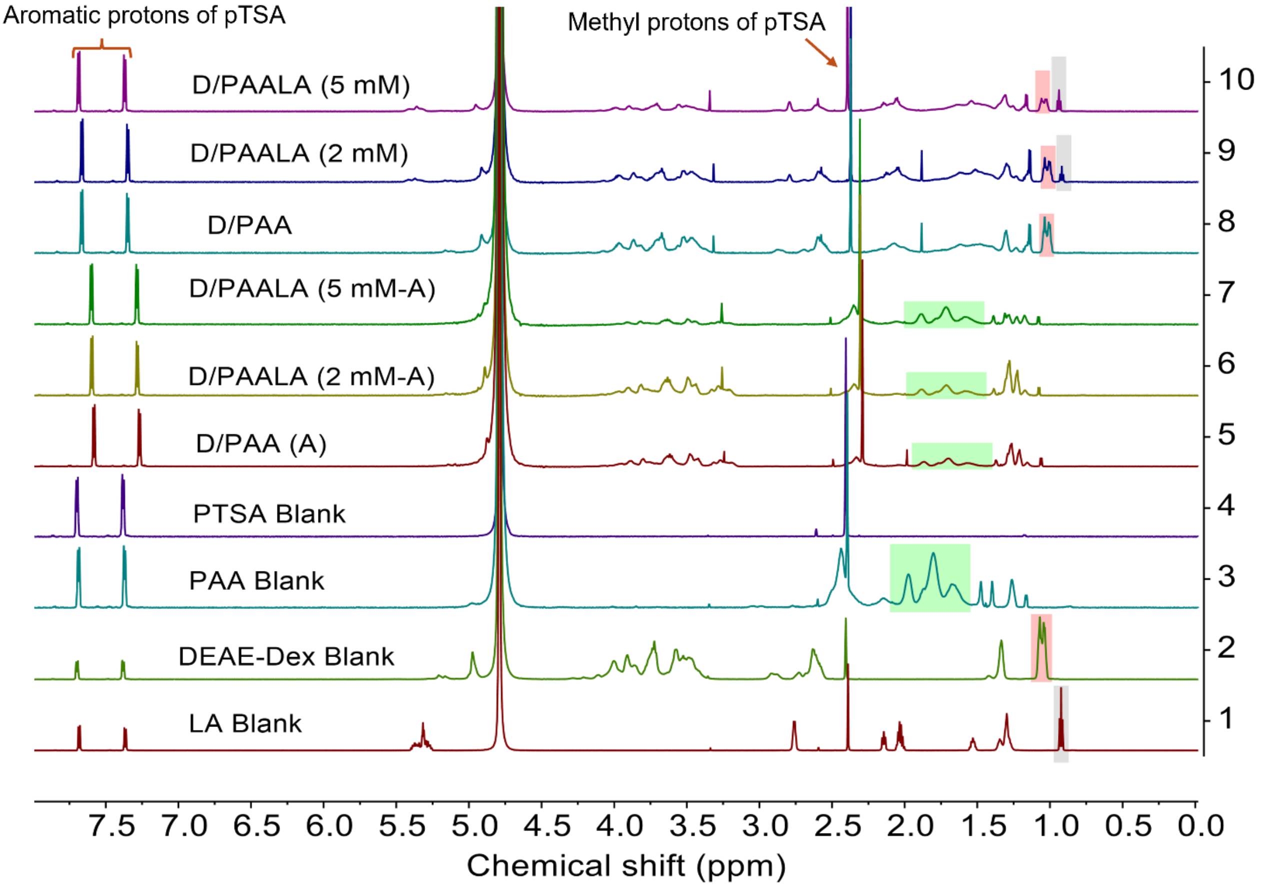

Figure A: *Plot showing NMR data of blank (LA, DEAE, PAA (A), and pTSA) and supernatant of three different coacervate systems without acidification and with acidification ('A' represents acidified supernatant) - DEAE/PAA (2.4 mM/ 17.3 mM), DEAE/PAA/LA (2.4 mM/ 17.3 mM/ 2 mM), and DEAE/PAA/LA (2.4 mM/ 17.3 mM/ 5 mM). Green shaded region for quantification of the PAA, the light red shaded region for quantification of the DEAE, and grey shaded region for quantification of the LA. The methyl and aromatic protons of pTSA (internal reference) are also labelled.*

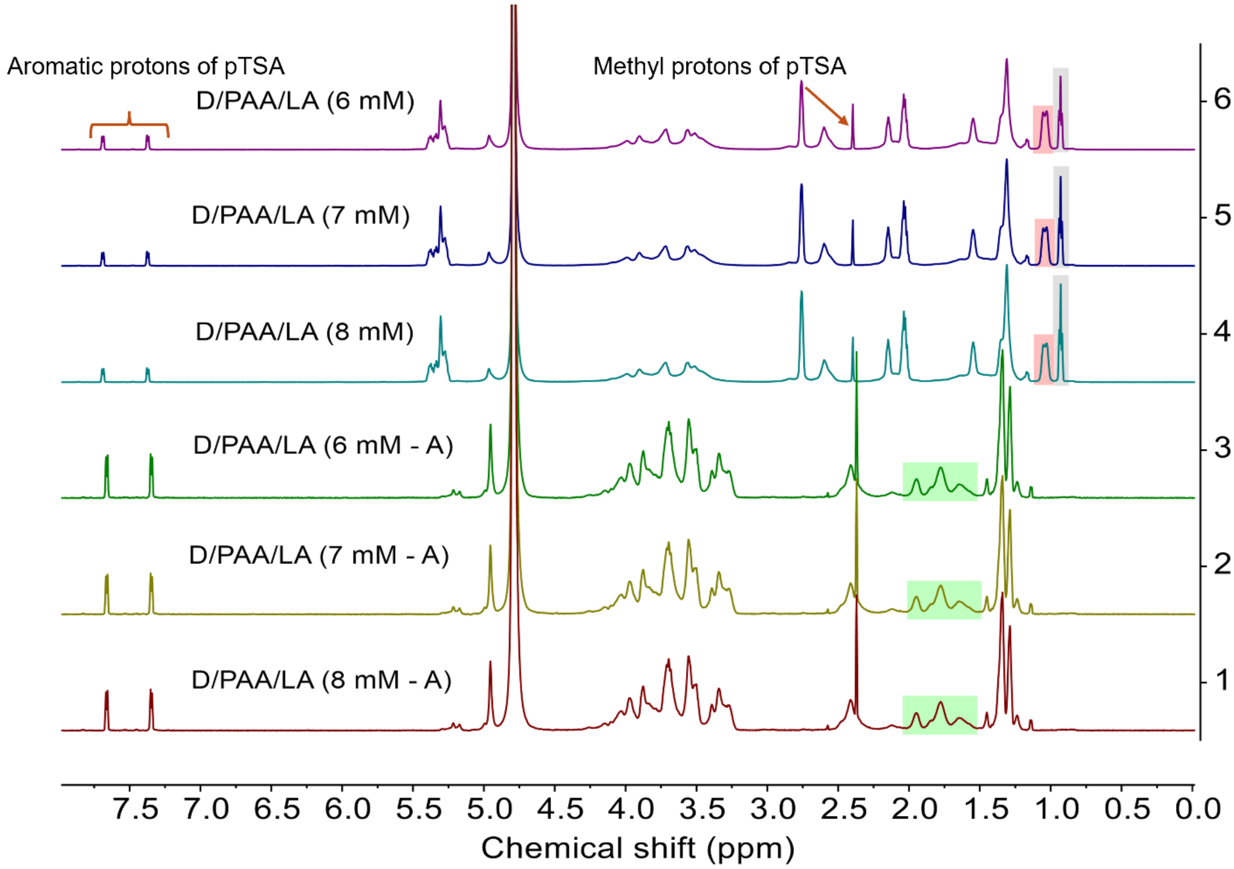

Figure B: *Plot showing NMR data of the supernatant phase of three different coacervate systems without acidification and with acidification ('A' represents acidified supernatant) - DEAE/PAA/LA (2.4 mM/ 17.3 mM/ 6 mM), DEAE/PAA/LA (2.4 mM/ 17.3 mM/ 7 mM), and DEAE/PAA/LA (2.4 mM/ 17.3 mM/ 8 mM). Green shaded region for quantification of the PAA, the light red shaded region for quantification of the DEAE, and grey shaded region for quantification of the LA. The methyl and aromatic protons of pTSA (internal reference) are also labelled.*

We performed NMR peak area analysis on solutions of DEAE, PAA and LA of different concentrations for calibration and showing the linearity of the increase in peak areas with concentration which is necessary for quantification. The table summarising the calibration data is shown below and the corresponding plot is shown in Figure C.

Table of peak area of different polymer and fatty acid concentrations for calibration:

DEAE [mM]	Peak area	PAA [mM]	Peak area	LA [mM]	Peak area
0.5	4.5	2	3.6	1	1.9
1	8.8	5	6.7	3	5.9
2	17	10	12.4	5	9.5
3	25.1	15	17.7	8	16.3
4	32.7	20	22.6	12	24.5

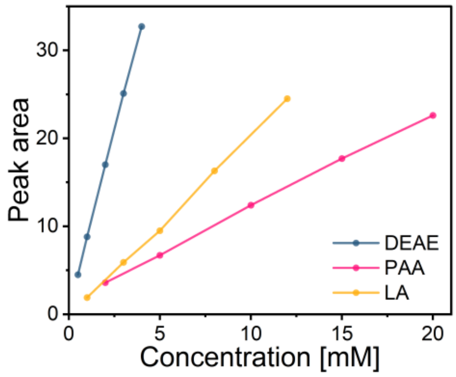

Figure C: *Plot showing the linear increase in NMR peak area of the selected protons with an increase in concentrations of DEAE, PAA, and LA.*

Effect of pH on displacement of PAA with LA

The pH was increased from 8.7 to 9.5 via addition of 30 µL of 0.1 M pH 9.8 carbonate buffer in 1 mL of LA/DEAE/PAA (5 mM/2.4 mM/17.3 mM) coacervate solution of pH 8.7, to check for displacement of PAA by LA on increasing pH. The coacervate solution at pH 8.7 and 9.5 was centrifuged, their supernatant was collected, and internal reference (pTSA) was added into it for NMR analysis. At pH 8.7, LA and DEAE were mostly present in the coacervate phase and when the pH was raised to 9.5, an increase in peak areas of PAA, DEAE and LA were noted (see table below and **Figure D**). This suggests that LA micelles in their low charged state (pH 8.7) are sequestered into the coacervate phase and are not able to displace PAA effectively. Upon raising the pH to 9.5, the increase in charge on the LA micelles enables better displacement of the PAA and partial displacement of DEAE via formation of soluble polyelectrolyte complexes.

Table of peak area on changing pH:

Name	DEAE peak area	LA peak area	PAA (after acidification) peak area
DEAE/PAA/LA (5 mM) pH 8.7	-	0.8	10.9
DEAE/PAA/LA (5 mM) pH 9.5	3.3	2.8	13.5

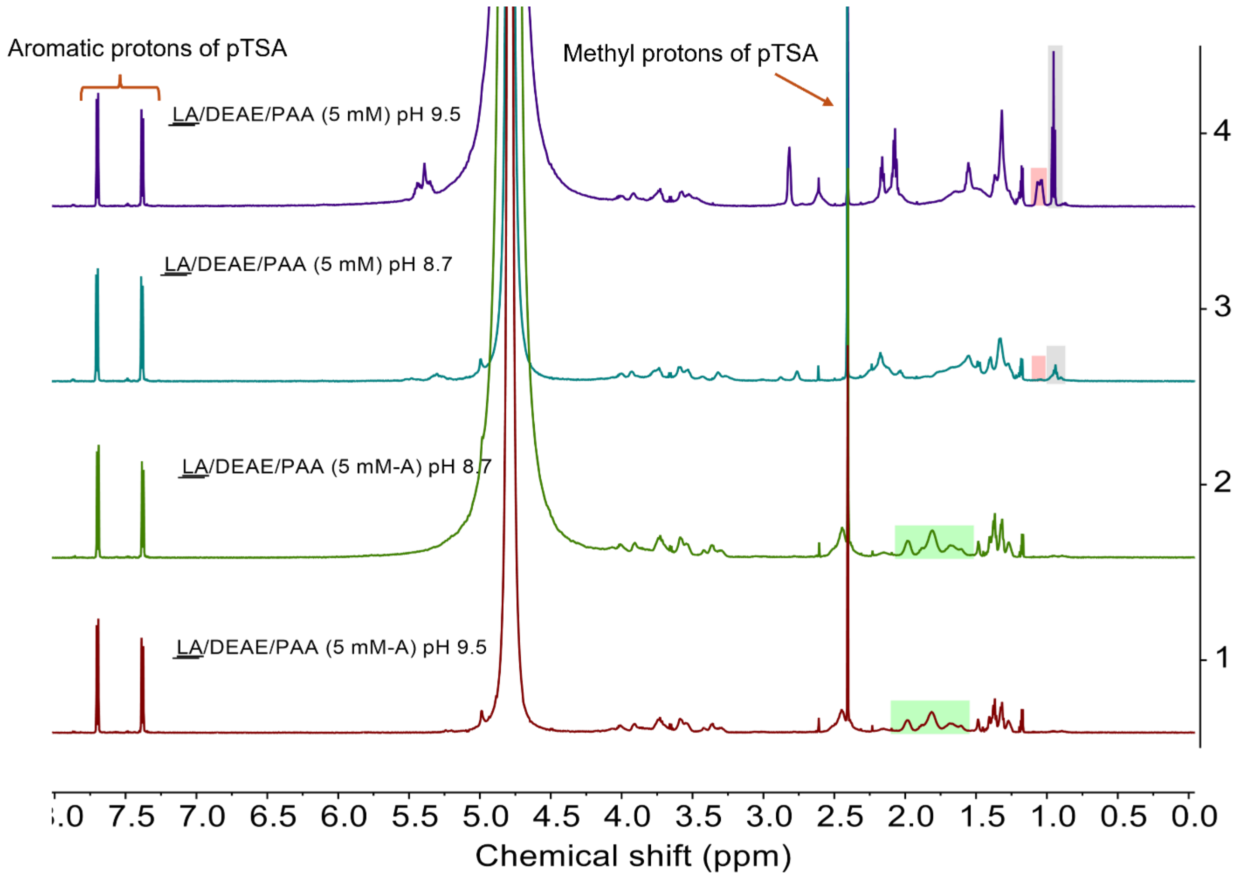

Figure D: *Plot showing NMR data of LA/DEAE/PAA (5 mM/2.4 mM/17.3 mM) at different pH to check the displacement of PAA with LA on increase in pH from 8.7 to 9.5 of the coacervate system, showing peak area for PAA was increasing in supernatant with increase in pH.*

Interestingly, when we mixed LA/DEAE/PAA (5 mM/2.4 mM/17.3 mM) coacervates with PDDA/PAA (10 mM/45 mM) coacervate droplets at pH 8.7, we observed complete wetting of the PDDA/PAA droplets by the LA/DEAE/PAA droplets. When the pH was raised to 9.5 by local addition of a carbonate buffer (20 µL of 0.1 M carbonate buffer, pH 9.8), we observed the vacuolization of the red LA/DEAE/PAA phase along with complete dewetting of the PDDA/PAA droplets (as shown in **Figure E**). The above results confirm that the switching of the coacervate composition of LA/DEAE/PAA upon raising the pH from 8.7 to 9.5 investigated via 1H NMR analysis, allowed it to go from wetting to dewetting of the PDDA/PAA droplets.

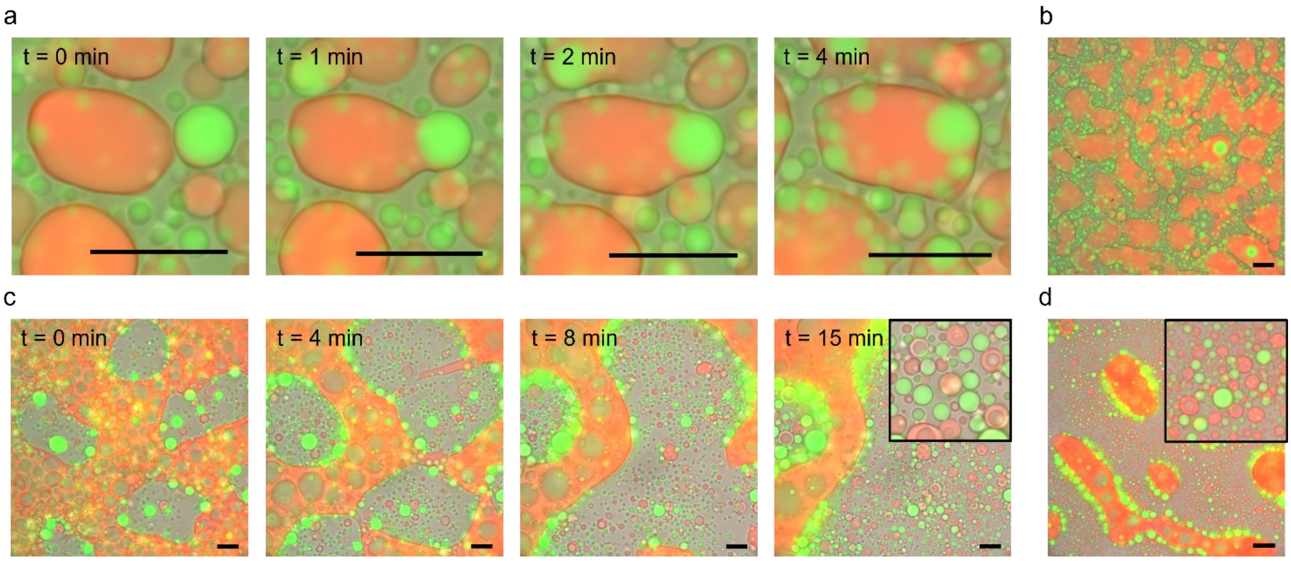

Figure E. a) Time lapse of brightfield/fluorescence microscopy images showing wetting of LA/DEAE/PAA (red droplets) (5 mM/2.4 mM/17.3 mM) coacervate droplets and PDDA/PAA (green droplets) (15 mM/45 mM) coacervate droplets at pH 8.7 and all over the viewing area all LA/DEAE/PAA coacervate droplets wetted PDDA/PAA coacervate droplets as shown in (**b**). After adding 20 μL of 0.1 M carbonate buffer, pH 9.8, locally rising pH of the solution to 9.5, showing dewetting of the PDDA/PAA and LA/DEAE/PAA coacervate droplets over time, followed by vacuolisation in the LA/DEAE/PAA coacervate phase as shown in (**c,d**). Inset showing dewetted droplets in (**c,d**). Scale bars are 20 μm in (**a-c**) and 50 μm in (**d**).

Note S2

Analysis of percentage overlap of droplets using MATLAB code

To aid the visualization of the dewetting process between the two phases of the multiphase coacervates, a MATLAB code was used to process the red/green channel fluorescence microscopy images individually to analyze the extent of wetting interactions between the DEAE-Dex/PAA phase (red channel) and the PDDA/PAA phase (green channel). We defined a parameter called "overlap percentage" which would characterize the amount of overlap between the red and green droplets within the microscopy images.

$$Overlap\% = \frac{area\ of\ overlap\ between\ red\ and\ green\ droplet}{area\ of\ green\ droplet} \times 100$$

For the case where the green droplets are completely inside the red droplets which would be the complete wetting condition, the green droplets should have 100% overlap with the red droplet. When the green droplet is entirely inside the red droplet still the area of overlap between the red and green droplet will not be 100 % since there will be no red fluorescence intensity where the green droplet is present. So, we used the '*imfill*' function to fill the holes of the red droplets and give 100% overlap. In the case where both droplets are completely dewetted, that is the green droplets are completely outside the red droplets, there is 0% overlap between the droplets. To follow the gradual wetting of the red droplets over the green droplets, the red droplet area was dilated using the '*imdilate*' function to detect the contact area as overlap % between the red/green droplets. As the green droplets go into the red droplets the contact area increases and the overlap % increases following the increase in wetting interactions between the droplets.

Briefly we discuss below how the MATLAB code analyses the overlap percentage from the fluorescence microscopy images -

The code starts with defining the input and output directories. We used '*padarray'* function to avoid boundary issues in following image processing steps. The images were separated into the red and green channels. The red and green channels were binarized and thresholded to detect the respective droplet areas. The next step is to find the edge of red droplets, which is necessary to get a more precise value of the overlap percentage. The '*edge*' function with the canny method was used to detect the red boundary, followed by the dilation of boundaries with an immediate function to make the red region more distinctive for better analysis. It is essential to fill the red boundaries and assume them as a single droplet to compare it with the green droplet for overlap. To separate the closely touching green droplets that can misjudged as single droplets, that '*watershed'* function is used to separate green regions that are touching each other to be analysed separately. Since individual green droplet need to be analysed to get average overlap percentage, '*bwconncomp*' function is used to identify all the green droplet in the entire image. once the individual green droplets are identified, the overlap percentage with red droplets are calculated for each green droplet. Further, the average overlap percentage of each image with standard deviation is calculated and saved in output directory folder. To visualize the average overlap percentages of green droplets in the image with red droplets, the red droplet was first colored as white areas for better visualization. The overlap percentage of each green droplet with red droplets was color-coded with a customized colormap that is a colour gradient of red, green to blue color showing 100 % and 50% to 0% overlap, respectively.

Supplementary videos

Movie S1: Brightfield and fluorescence microscopy video showing wetting of LA/DEAE/PAA coacervate droplets and PDDA/PAA coacervate droplets at pH 8.7, and after addition of 20 μL of 0.1 M carbonate buffer, pH 9.8, causes droplets to dewet each other due to a rise in pH. The video of dewetting and wetting was shown at x70 of real time.

Movie S2: Brightfield and fluorescence microscopy videos of DEAE/PAA/PDDA multiphase coacervate droplets showing dewetting (left, brightfield) upon addition of 2.5 mM OA at pH 10 and wetting (right, brightfield/fluorescence) of green PDDA/PAA droplets by red OA/DEAE/PAA droplets upon pH drop due to atmospheric CO_2 dissolution in the sample. The videos of dewetting and wetting are displayed at x20 and x100 of real time, respectively.

Movie S3: Green/red channel fluorescence microscopy videos of DEAE/PAA/PDDA multiphase coacervate droplets showing dewetting (left) upon addition of 2.5 mM LA at pH 9.3 and wetting (right) of green PDDA/PAA droplets by red LA/DEAE/PAA droplets upon pH drop due to atmospheric CO_2 dissolution in the sample. The videos of dewetting and wetting are shown at x24 and x205 of real time, respectively.

Movie S4: MATLAB processed fluorescence microscopy video showing multiple cycles of reconfiguration of DEAE/PAA/PDDA multiphase coacervate droplets in the presence of 2.5 mM LA upon local addition of aliquots of carbonate buffer (4 μL, pH 10, 50 mM) over a large area. Here, the PDDA/PAA droplets are color coded with respect to their overlap % with LA/DEAE/PAA droplets (false colored in white). Initially, the multiphase coacervate droplets are in wetting condition at pH 8.3 with the PDDA/PAA droplets colored red (100 % overlap), but after addition of buffer adjacent to the viewing area, the multiphase droplets dewetted with the PDDA/PAA droplets colored blue (0% overlap), and then the pH dropped gradually via atmospheric CO_2 dissolution, causing the droplets to wet. Similarly, four successive cycles were observed, after which the system fatigued, and the droplets remained dewetted (the pH of the system increased above 9). The video of dewetting and wetting was shown at x413 of real time.

Movie S5: Fluorescence microscopy and color-coded composite video showing multiple cycles of dewetting-wetting (in presence of 2.5 mM LA) of the multiphase coacervates within a small area. The video of dewetting and wetting was shown at x413 of the real time.

Movie S6: Brightfield microscopy video showing dewetting-wetting (in presence of 2.5 mM OA) of the multiphase coacervates. Initially, started at pH 9 where droplets were in wetted state and after addition of 4 μL of buffer (sodium carbonate, pH 10, 50 mM) which causes dewetting of the droplets and with time pH dropped which leading wetting of the droplets. In the same way, three consecutive cycles were recorded, after that fatigue was observed in the system. The video of dewetting and wetting was shown at x560 of the real time.

Movie S7: Brightfield microscopy video showing vacuolisation of the outer phase in multiphase droplets as the basic pH front propagates outwards from the urease microgel beads through the multiphase coacervate droplet population. The video is shown at x100 of the real time.

Movie S8: MATLAB processed fluorescence microscopy video showing a cascade of reconfiguration (dewetting) of the multiphase coacervate (in the presence of 2.5 mM LA) droplets (starting from pH 8.3) after addition of urease beads which propagate a basic pH front. The video is shown at x710 of the real time.

Supplementary Figures

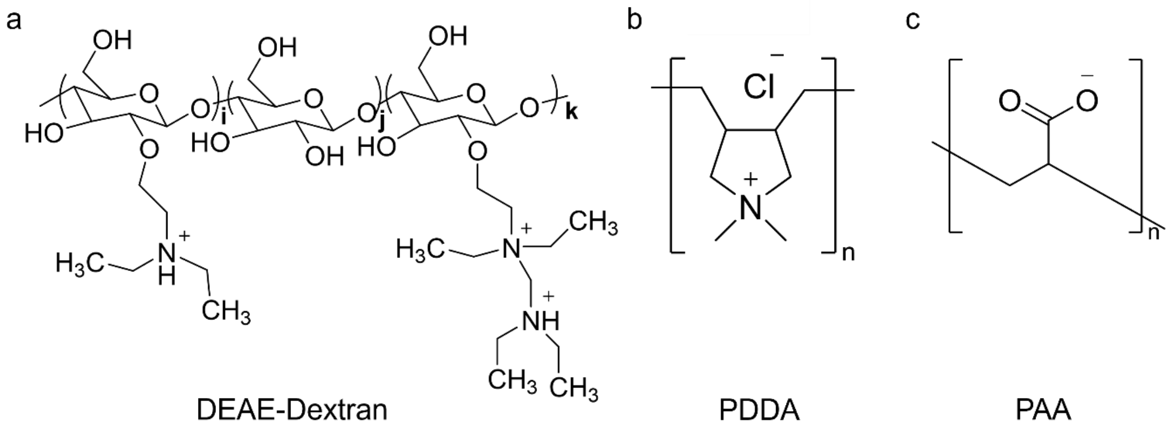

Figure S1. (a-c) Molecular structures of polyelectrolytes used in the formation of the DEAE/PAA/PDDA multiphase coacervates.

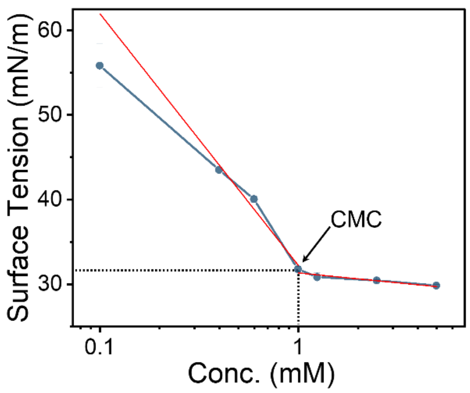

Figure S2. Plot showing the CMC of linolenic acid (LA) calculated via Drop shape analyser instrument by measuring of surface tension of different concentrations of LA showing drop in the value of surface tension and becoming constant after certain point. The intersection points of both slope give the value of CMC~ 1 mM.

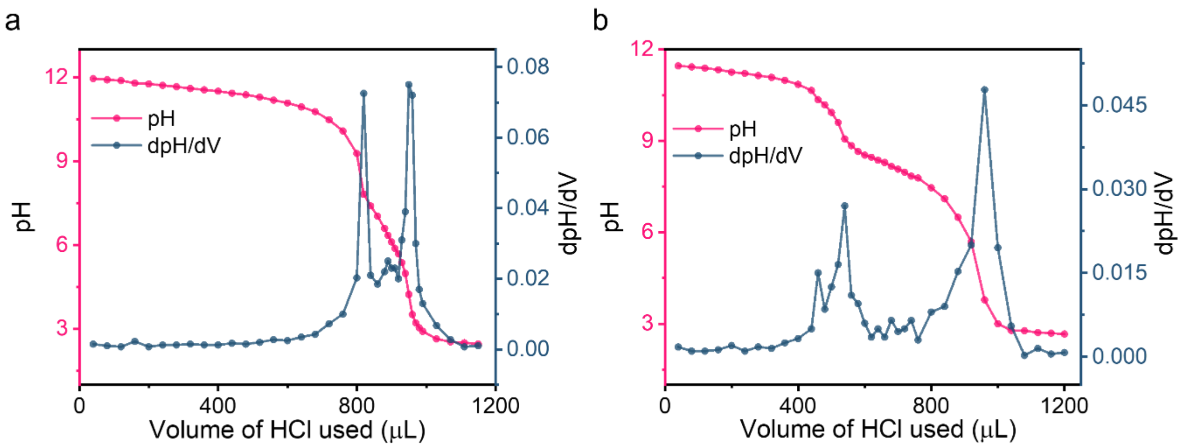

Figure S3. (a) Plot showing pH titration data of 0.5 mM LA with 0.145 N HCl solution shown as a pink trace. The plot of dpH/dV (blue trace) shows two equivalence points – first, corresponds to the neutralization of NaOH (at 0.82 mL), and the second corresponds to the titration of carboxylate of LA at 0.95 mL, for which pK_a corresponds to ~6.4. **(b)** Plot showing pH titration data of 1.5 mM LA with 0.145 N HCl solution shown as a pink trace. The plot of dpH/dV (blue trace) shows two equivalence points – first, corresponds to the neutralization of NaOH (at 0.54 mL), and the second corresponds to the titration of carboxylic acid of LA at 0.96 mL, for which pK_a corresponds to ~7.8.

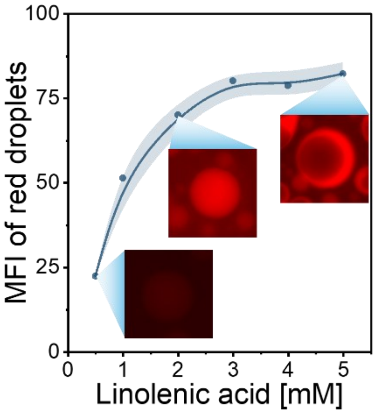

Figure S4. Plot showing an increase in red fluorescence intensity in DEAE/PAA (2.4/17.3 mM) coacervate droplets with increase in LA concentration (0.5–5) mM at pH 9.3, inset showing red fluorescence microscopy images.

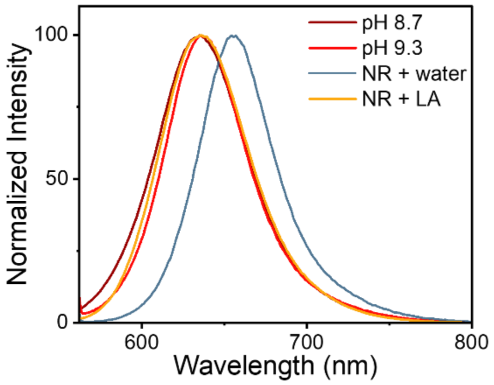

Figure S5. Plot showing emission spectra of Nile red (3.3 μM) in water (control, blue), in aqueous solution of LA (3 mM, yellow) and in the LA/DEAE/PAA coacervate phase (3/2.4/17.31 mM), shades of red) at pH 9.3 and 8.7. Nile red emission is sensitive to the polarity of its local environment. The emission λ_{max} of nile red in LA solutions (636 nm) is blue shifted from its emission λ_{max} in water (656 nm) indicating the nile red in LA solutions is present mostly within micelles of LA. The similarity of the emission spectra of LA solutions and the coacervate phase (λ_{max}= 636 nm) suggests that the nile red containing micelles of LA are present in the coacervate phase.

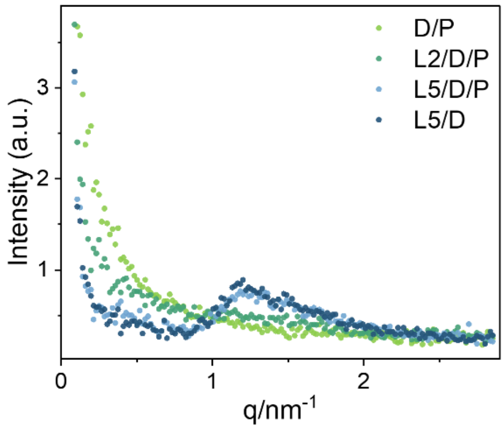

Figure S6. SAXS profiles of DEAE/LA (L5/D), DEAE/PAA coacervate phase with no LA added (D/P) and with 2 mM (L2/D/P) and 5 mM (L5/D/P) LA added. Upon incorporating LA into the DEAE/PAA coacervate phase, a peak appears at ~1.20 nm^{-1} which increases in intensity with rise in LA content and this peak is also present in DEAE/LA coacervate phase. This broad peak suggests weak ordering of LA micelles within the coacervate phase.

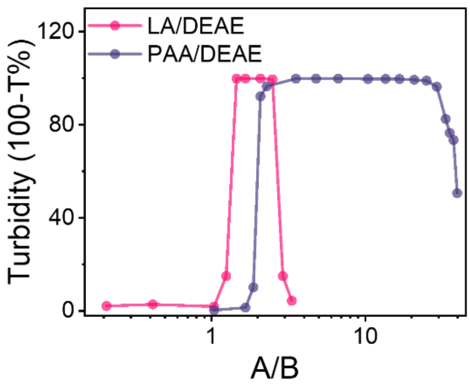

Figure S7. (a) Plot of turbidity against molar/monomer ratio of polyanion/polycation (A/B) showing the stoichiometry range of existence of LA/DEAE and PAA/DEAE coacervates. The concentration of DEAE-Dex was fixed at 2.4 mM in studies with LA and 4.8 mM in studies with PAA.

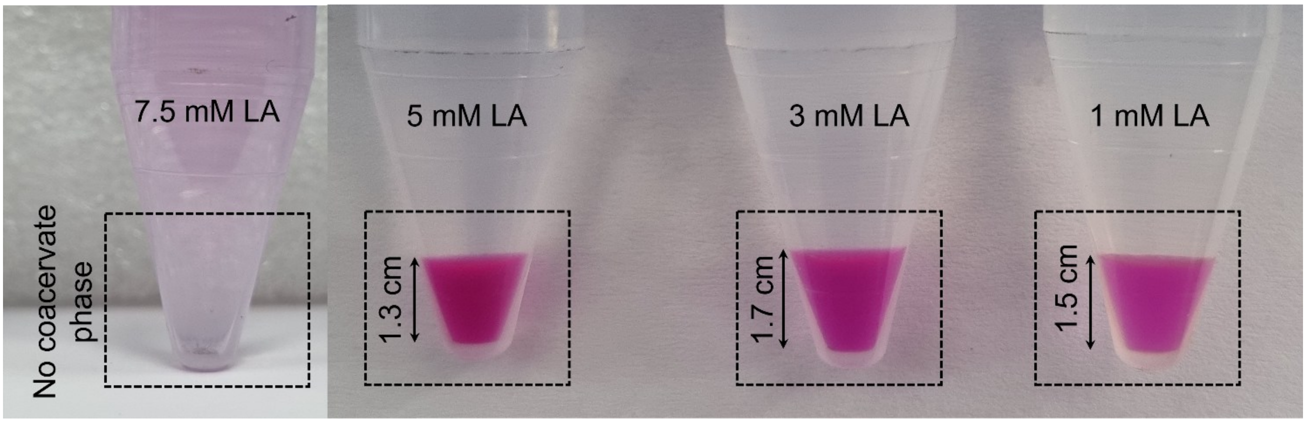

Figure S8. Photograph showing centrifuged sample vials containing DEAE/PAA (2.4 mM/17.3 mM) with different LA concentrations (1,3, 5, and 7.5 mM). On increasing LA concentration, the volume of the coacervate phase increased slightly at 3 mM LA and then decreased at 5 mM LA and at 7.5 mM LA coacervate phase completely disappeared.

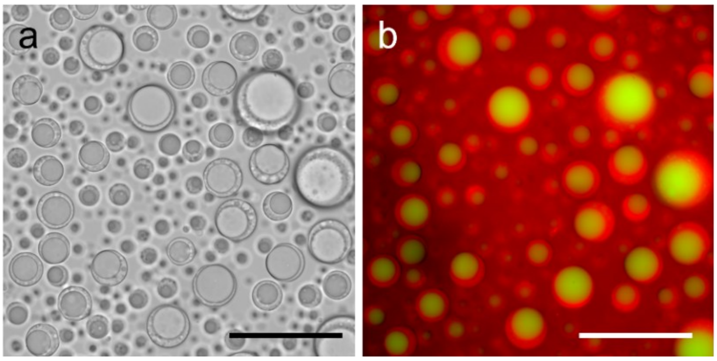

Figure S9. (a) Brightfield and **(b)** fluorescence microscopy images of DEAE/PAA/PDDA multiphase coacervates showing an outer DEAE/PAA phase labelled using RITC-DEAE and inner PDDA/PAA phase stained using green-fluorescent Pyranine. Scale bars are 50 µm.

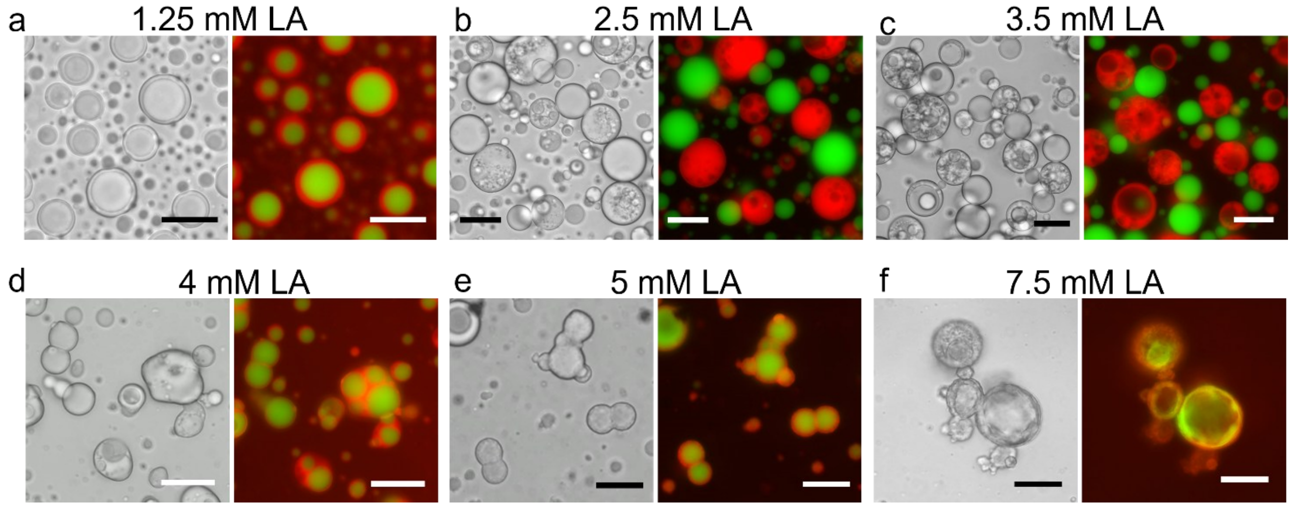

Figure S10. Red/green channel fluorescence (right) and brightfield (left) microscopy images showing multiphase coacervate droplets (DEAE/PAA/PDDA (2.2/40/10 mM)) with **(a)** 1.25 mM, **(b)** 2.5 mM, **(c)** 3.5 mM, **(d)** 4 mM, **(e)** 5 mM, and **(f)** 7.5 mM of Linolenic acid (LA) added to the sample along with nile red (9 µM, red channel) and pyranine (9 µM, green channel) at pH 9.3. At 2.5 mM LA, the two phases dewetted each other and with further increase in LA concentration (3.5 mM), the LA/DEAE/PAA phase (red droplets, Nile red) started to vacuolise. At 4 mM LA, LA started to interact with PDDA/PAA coacervate droplets and very less of LA/DEAE/PAA phase was present. With 5 mM LA, the LA/DEAE/PAA phase completely dissolved, and LA started to interact with PDDA/PAA coacervate to form an outer layer rich in LA (polyelectrolyte complex of LA/PDDA) preventing fusion between droplets and further increase in LA to 7.5 mM vacuolised the PDDA/PAA coacervate droplets leaving a thin layer of the coacervate phase inside. Scale bars are 20 µm.

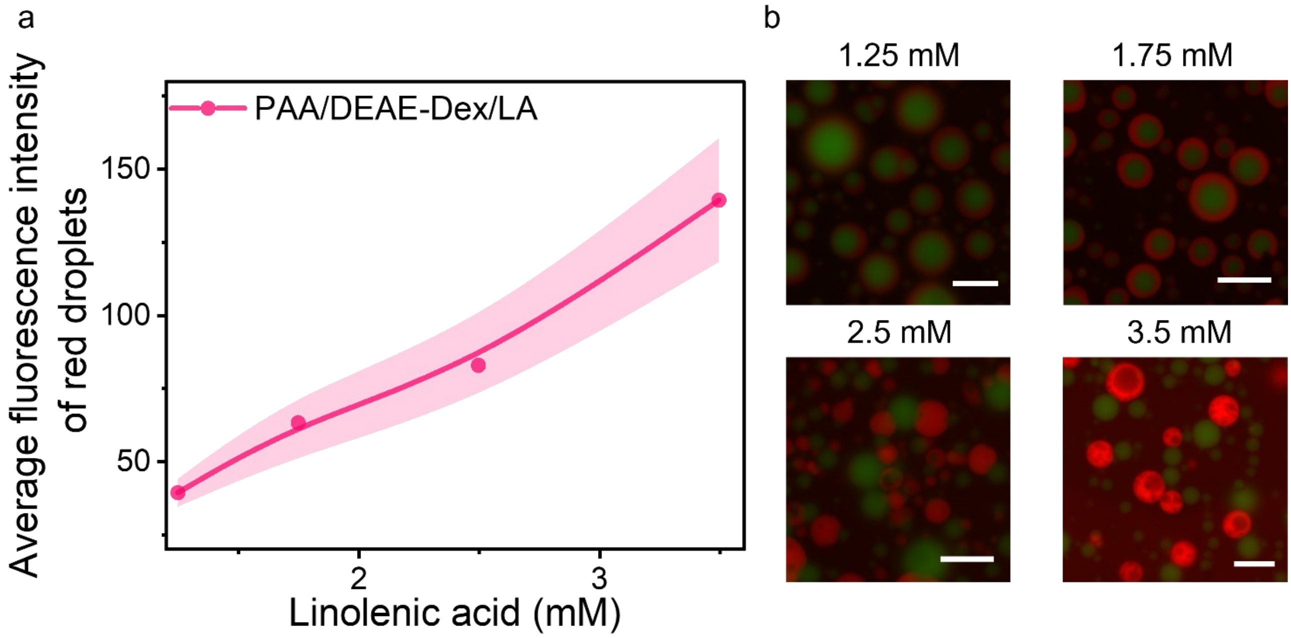

Figure S11. (a) Average fluorescence intensity plot showing increase in fluorescence intensity (due

to Nile red, 2.5 μM) of the red droplets with increase in LA concentration highlighting more incorporation of LA in outer phase upto 3.5 mM LA, which corresponds to red/green fluorescence microscopy images (every image captured at same exposure time of 18.69 ms in red channel) at different concentrations of LA as shown in **(b).** Scale bars are 20 μm.

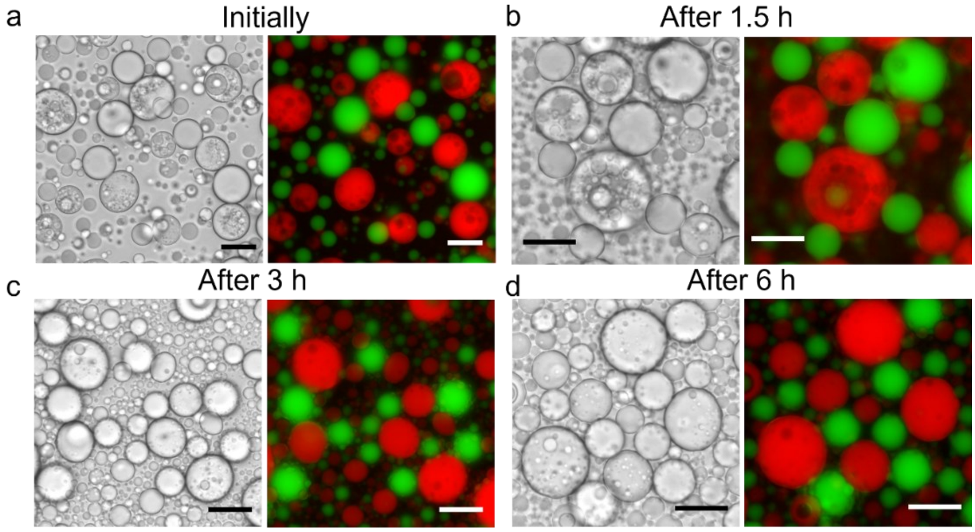

Figure S12. Red/green channel fluorescence (right) and brightfield (left) microscopy images **(a-d)** showing no wetting interactions between the green PDDA/PAA droplets (9 μM pyranine) and the red LA/DEAE/PAA droplets (9 μM, nile red) formed upon addition of 2.5 mM LA, even after 6 hours, when the pH was maintained by addition of carbonate buffer (0.05 M, pH 9.3). Scale bars are 20 μm.

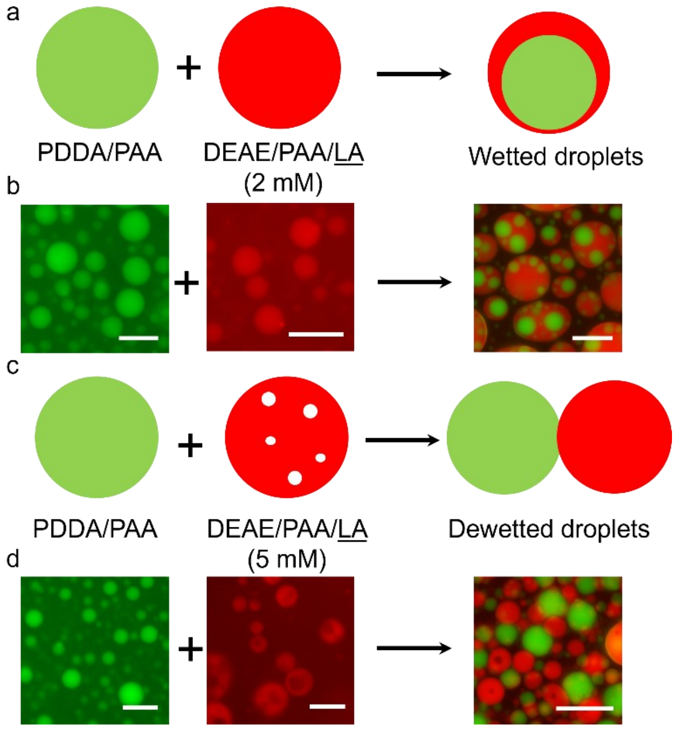

Figure S13. (a,c) Schematic showing mixing of PDDA/PAA and LA/DEAE/PAA coacervates showing wetting interactions at 2 mM LA and non-wetting condition at 5 mM LA, between the two coacervate droplets. **(b,d)** Fluorescence microscopy images of LA/DEAE/PAA (2/2.4/17.3 mM) and LA/DEAE/PAA (5/2.4/17.3 mM), red droplets (9 μM nile red) mixed with PDDA/PAA (15 mM/45 mM) green droplets (9 μM pyranine), showing at 2 mM LA both coacervates were wetting each other while

at 5 mM LA, both coacervate droplets shows non-wetting condition and in both cases pH was maintained to 9.3. Scale bars are 20 μm.

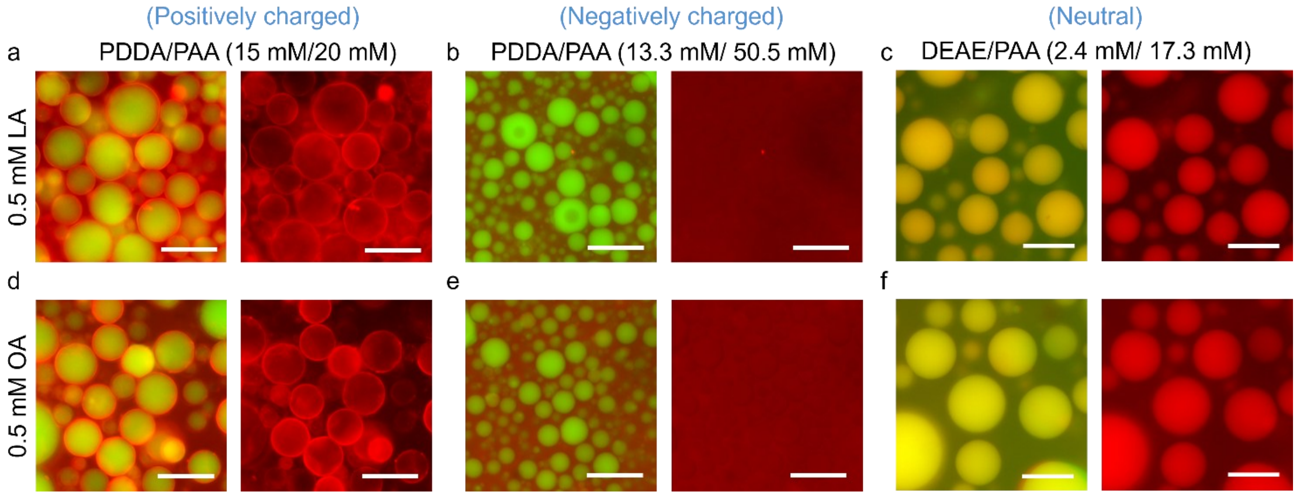

Figure S14. **(a-f)** Red/green (left) and red (right) fluorescence microscopy images showing interactions of 0.5 mM of LA and OA with **(a,d)** positively charged (+17.8 mV) PDDA/PAA (15 mM/20 mM), **(b,e)** negatively charged (-32.8 mV) PDDA/PAA (13.3 mM/50.5 mM), and **(c,f)** neutral (+0.8 mV) DEAE/PAA (2.4 mM/17.3 mM) coacervate droplets showing surface adsorption of LA/OA only in case of positively charged PDDA/PAA coacervate droplets, showing charged based interaction of LA/OA with coacervate droplets. In case of DEAE/PAA, LA/OA gets sequestered inside the coacervate droplets. Note: Both PDDA/PAA and DEAE/PAA coacervate droplets were stained with pyranine, and LA/OA were stained with Nile red. Scale bars are 20 μm.

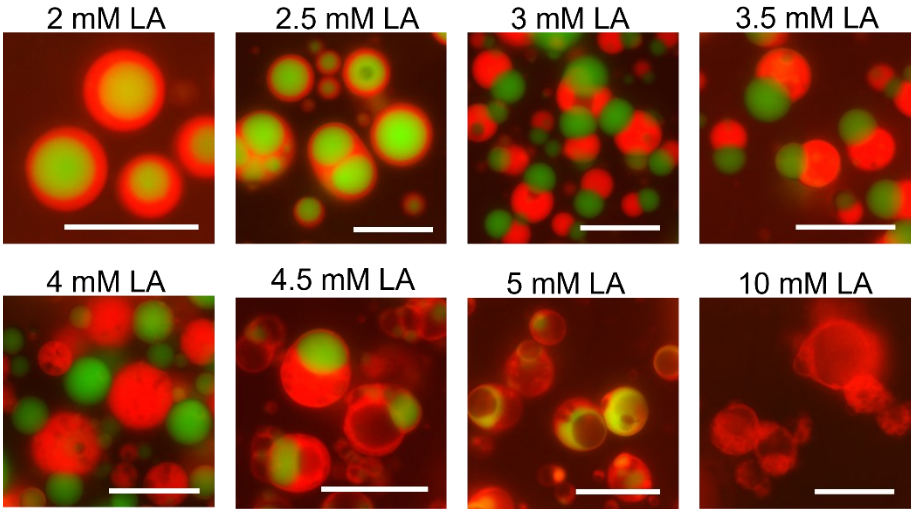

Figure S15. Red/green channel fluorescence microscopy images showing the effect of different concentrations of LA on DEAE/PAA/PDDA (2.21/40/10 mM) multiphase coacervate droplets at pH 8.7. At 2 mM and 2.5 mM LA, outer LA/DEAE/PAA coacervate wetted the PDDA/PAA core coacervate droplets, and with increasing the LA concentration to (3-3.5) mM LA, partial dewetting was observed, and at 4 mM LA, two coacervate phases dewetted each other. At 4.5 mM and 5 mM LA, LA started to interact with PDDA/PAA coacervate droplets, and at 5 mM LA, the PDDA/PAA coacervate phase started to vacuolize. With 10 mM LA, the LA/DEAE/PAA phase completely dissolved, and LA started to interact with PDDA to form an LA/PDDA shell droplet, dissolving PDDA/PAA coacervate phase. Scale bars are 20 μm.

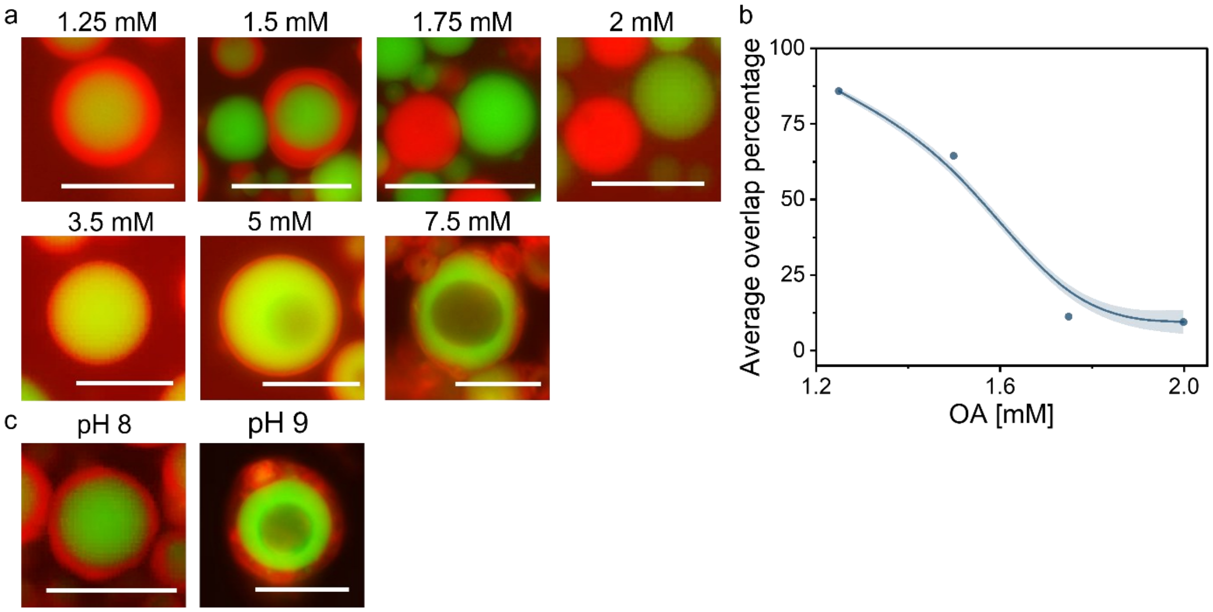

Figure S16. **(a)** Red/green channel fluorescence microscopy images showing DEAE/PAA/PDDA multiphase coacervates (2.21/40/10 mM) upon addition of different amounts of oleate, viz, from 1.25 mM to 7.5 mM at pH 10 along with nile red (9 μM, red channel) and pyranine (9 μM, green channel). With 1.25 mM OA, the micelles of OA (CMC for OA is 0.086 mM) are sequestered into the outer DEAE/PAA phase as indicated by the red fluorescence in the outer phase coming from the nile red present within the OA micelles. At 1.5 mM OA, dewetting started where (30-40)% droplets were dewetted and rest droplets staying in wetting state which is calculated via average overlap percentage and at 1.75 mM OA, the OA enriched DEAE/PAA phase and lead to complete dewetting of the PDDA/PAA droplets and at 3.5 mM OA and 5 mM OA, the OA/DEAE/PAA phase completely dissolved, and LA started to interact with PDDA/PAA coacervate to form an outer layer rich in OA (polyelectrolyte complex of OA/PDDA) preventing fusion between droplets. On further increase in OA concentration to 7.5 mM, OA started to vacuolise the PDDA/PAA coacervate droplets forming outer layer of OA/PDDA polyelectrolyte complexes. **(b)** The dewetting transition from 1.25 mM OA to 2 mM OA was calculated via MATLAB image analysis using average overlap percentage. **(c)** When 1.75 mM OA was added at pH 8 and pH 9, no dewetting was observed rather only sequestration of the OA into the outer DEAE/PAA phase was observed at pH 9 and at pH 8, formation of OA-rich droplets was observed in the outer phase. Scale bar is 20 μm.

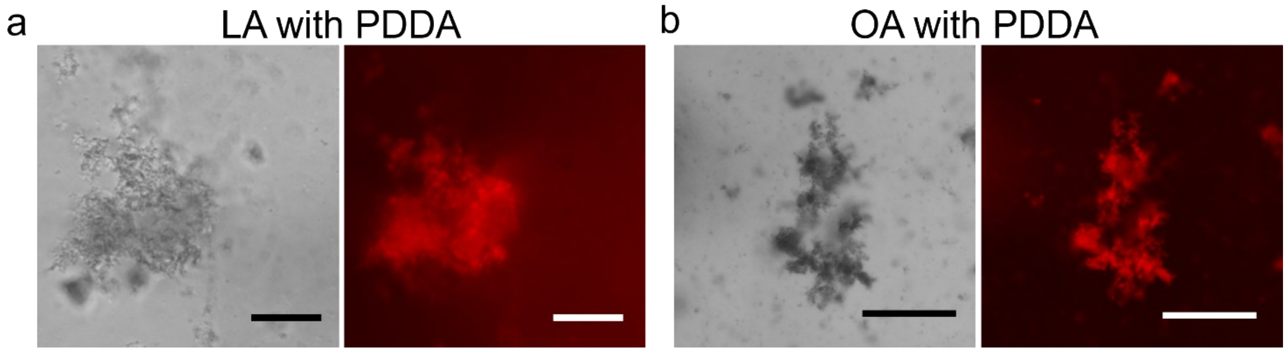

Figure S17. Brightfield (left) and fluorescence (right) microscopy images showing formation of

precipitates upon mixing **(a)** LA (2.5 mM) and **(b)** OA (2.5 mM) with PDDA (10 mM). Nile red (9 µM) was added for staining LA assemblies. The experiments with OA and LA were carried out at pH 10 and 9.3, respectively. Scale bars in (a) and (b) are 20 and 100 µm, respectively.

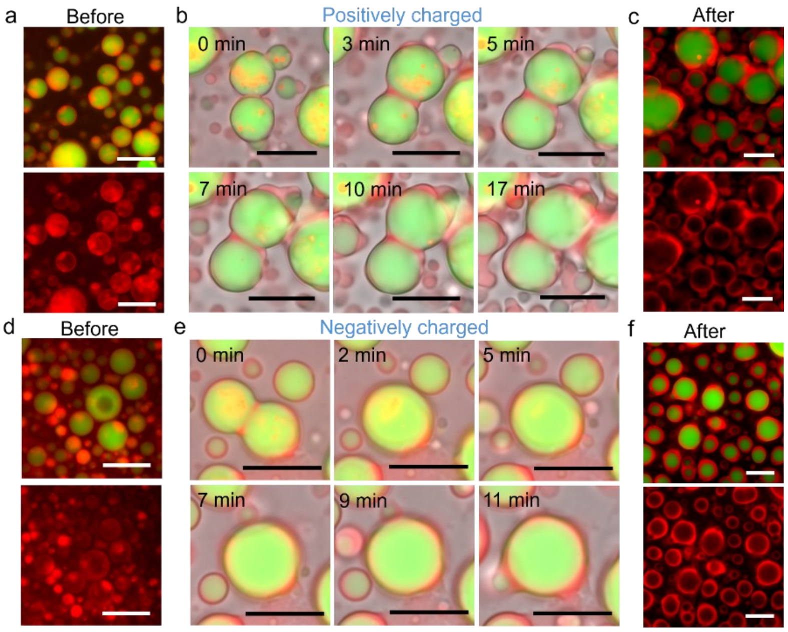

Figure S18. **(a,c,d,f)** Red-green channel (top) and red channel (bottom) fluorescence microscopy images showing LA (1.8 mM) interacting with **(a)** positively charged PDDA/PAA (16.4 mM/21.8 mM; +18 mV) droplets and **(d)** negatively charged PDDA/PAA (8.7 mM/60 mM; -38 mV) droplets, forming aggregates at the interface as well as internalizing inside coacervate droplets and **(c,f)** showing the disappearance of PDDA/LA aggregates and appearance of DEAE/PAA phase containing LA over a large area. Notably, less aggregates were formed at the interface of negatively charged PDDA/PAA droplets in comparison to the positively charged droplets but regardless the PDDA/LA aggregates entered both droplets suggesting that the interfacial aggregates were not functioning as a membrane. **(b,e)** Time lapse of brightfield/fluorescence (red-green channel) microscopy images showing the changes upon adding 1.2 mM DEAE into the PDDA/PAA coacervate with PDDA/LA aggregates. The aggregates slowly disappear and the LA is uptaken by the outer DEAE/PAA phase formed which illustrates the preferential interaction of LA with DEAE.[#] Scale bars are 20 µm.

#Preferential interaction of LA with DEAE may be attributed to its flexibility (smaller electrostatic contribution to persistence length due to low charge density) and ability to form hydrogen bonds with uncharged LA head groups as well.[10–12]

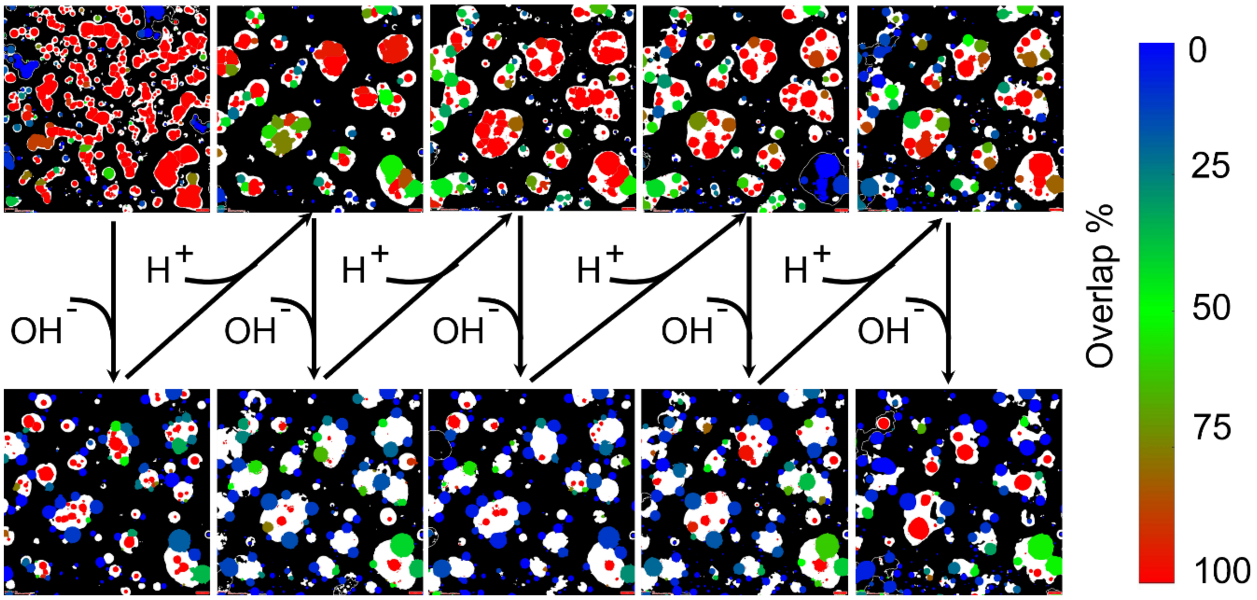

Figure S19. Processed fluorescence microscopy images with the LA/DEAE/PAA phase shown in white and the PDDA/PAA droplets color coded (color scale – right) according to their overlap %, showing four cycles of dewetting and wetting (in presence of 2.5 mM LA) upon local addition of 4 µL of buffer (carbonate buffer, pH 10) and subsequent acidification by atmospheric CO_2 dissolution. After four successive cycles, in the 5th cycle, the droplets stayed dewetted because the local pH of the system possibly stayed above pH 9, preventing the droplets from wetting.

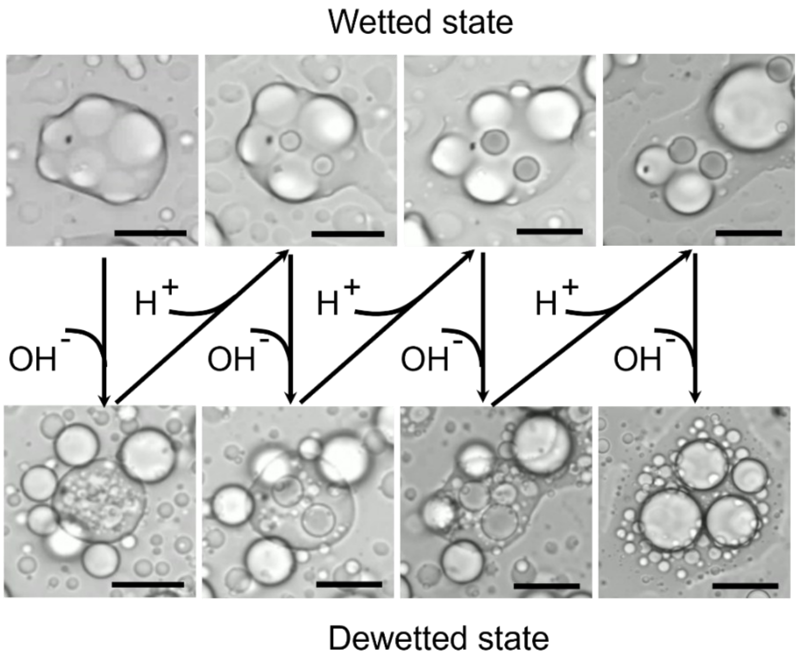

Figure S20. Sequence of brightfield microscopy images showing the dewetting and wetting cycles (three cycles) in DEAE/PAA/PDDA (2.21/40/10 mM) multiphase coacervates in the presence of 2.5 mM OA, induced by repeated local addition of carbonate buffer (4 µL, pH 10) and subsequent acidification by atmospheric CO_2 dissolution. After three cycles, further addition of 4 µL aliquots of buffer did not cause dewetting probably due to increase in local ionic strength due to repeated addition of buffer. Scale bar is 20 µm.

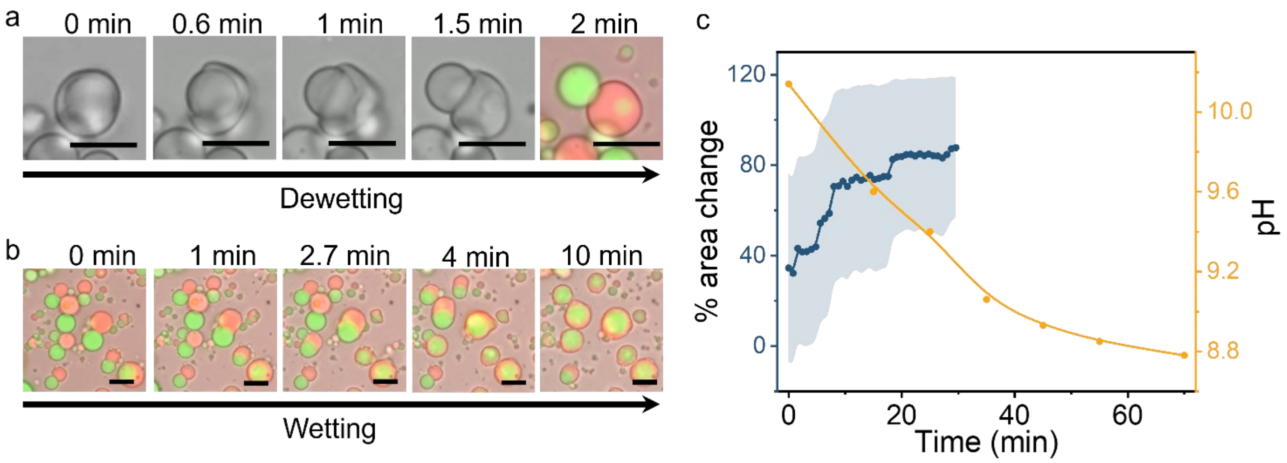

Figure S21. Time lapse of brightfield/fluorescence microscopy images showing the **(a)** dewetting of the DEAE/PAA/PDDA (2.21/40/10 mM) multiphase coacervate droplets following the addition of 2.5 mM oleic acid (OA) at pH 10 and **(b)** subsequent wetting of the PDDA/PAA droplets (green, 9 µM pyranine) by the red OA/DEAE/PAA droplets (red, 9 µM Nile red) due to pH drop caused by atmospheric CO_2 dissolution in the sample. **(c)** Plot showing increase in average overlap percentage indicating increased wetting interactions between the red and green droplets. The drop in pH due to atmospheric CO_2 dissolution in the sample causes a decrease in charge density of OA micelles (pK_a = 9.85) and their interaction with the DEAE/PAA phase which triggers the wetting process. The pH axis was a separate experiment where manually pH was measured in the solution (in imaging petridish) with longer time intervals. Scale bars are 20 µm.

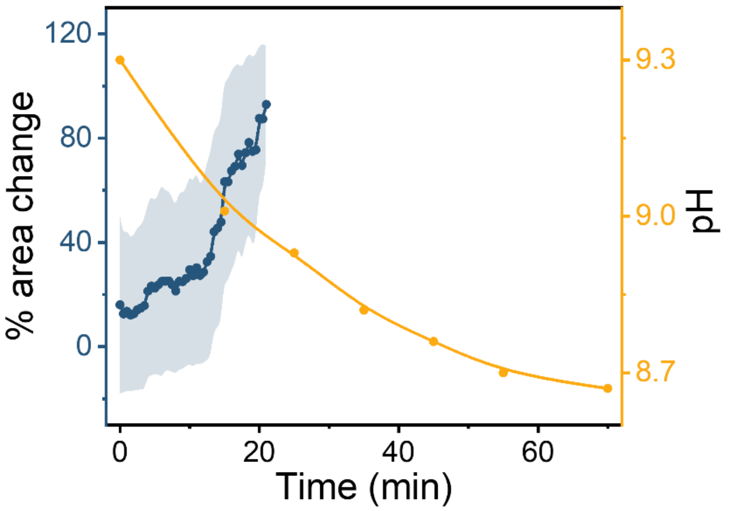

Figure S22. Plot showing increase in average overlap percentage indicating increased wetting interactions between the red LA/DEAE/PAA droplets and green PDDA/PAA droplets, the gradual dissolution of atmospheric CO_2 into the sample drops the pH, which reduces the interaction of LA with DEAE leading increased wetting interactions and formation of multiphase coacervate droplets. The pH axis was a separate experiment where manually pH was measured in solution (in imaging petridish) with longer time intervals.

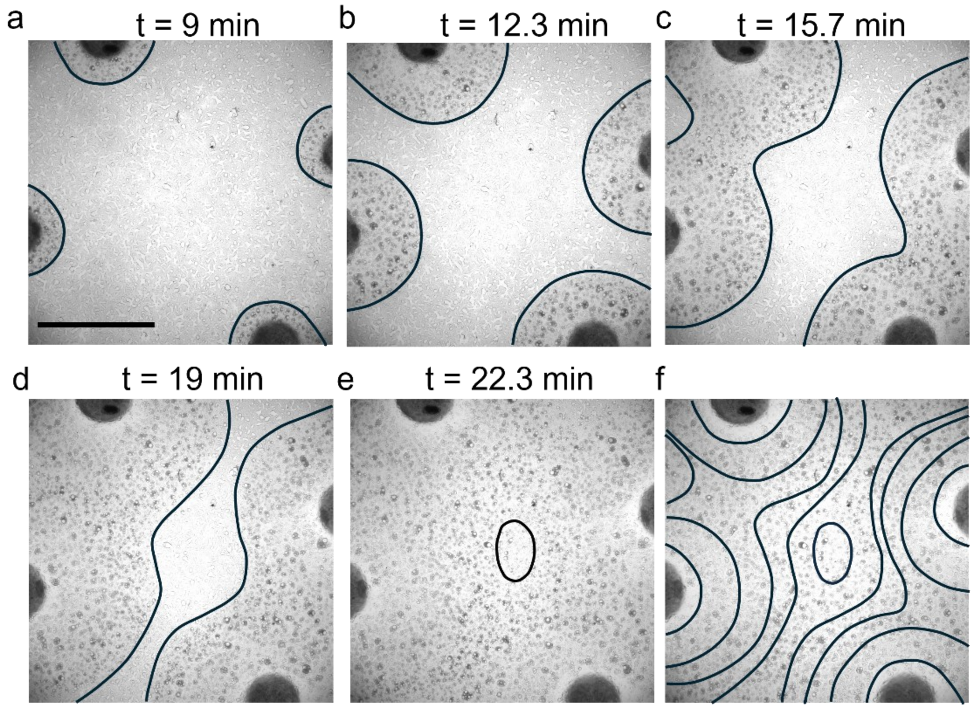

Figure S23. Time sequence of brightfield microscopy images showing propagation of coacervate response front (highlighted via black lines) visualized by the vacuolisation in outer LA/DEAE/PAA coacervate phase which is regulated by basic pH front propagated by the urease gel beads **(a-e). (f)** The lines demarcating the region containing droplets with vacuolized outer phase are overlayed in a single image taken at t = 22.3 min. Scale bar is 1 mm.

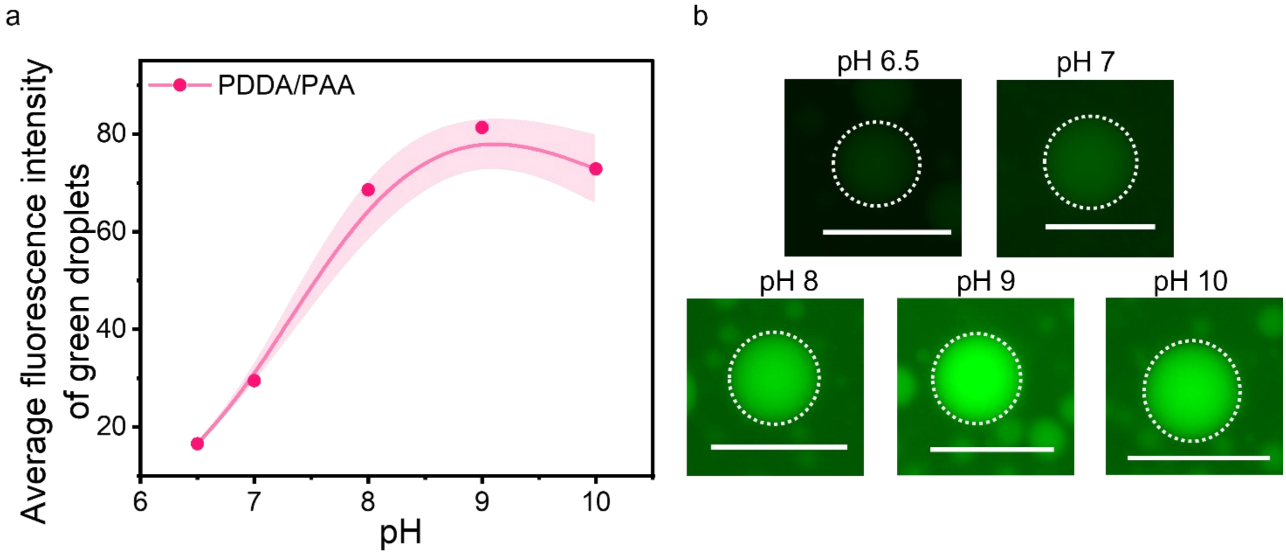

Figure S24. (a) Plot showing average green fluorescence intensity of pyranine (1 mM) in PDDA/PAA (15/45 mM) coacervate droplets showing increase in fluorescence intensity with pH and the corresponding fluorescence microscopy images are shown in **(b)**. Scale bars are 20 µm.

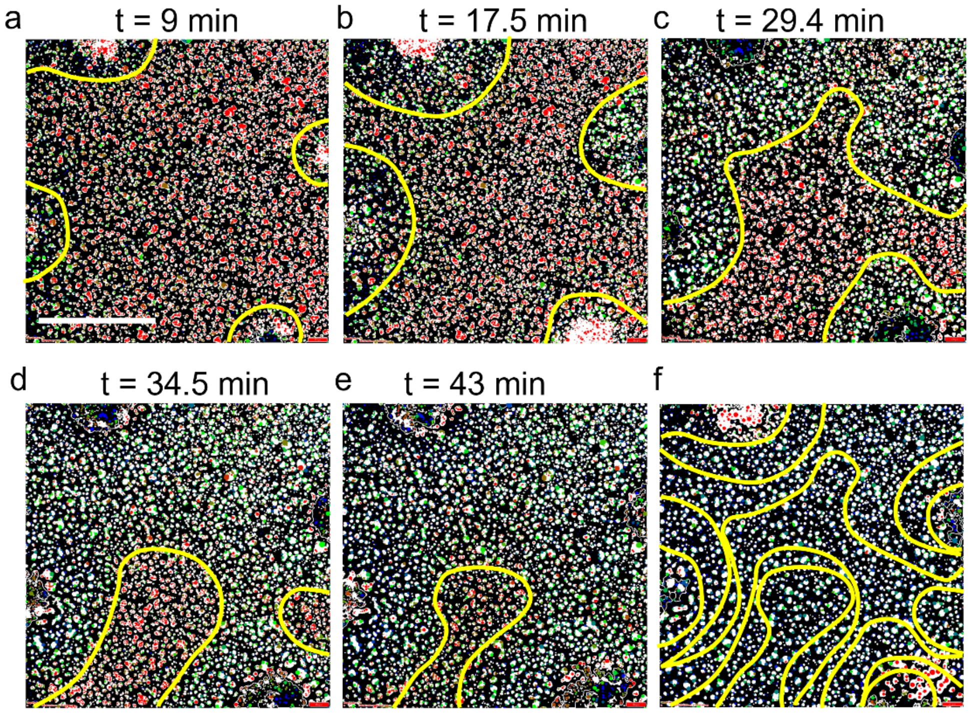

Figure S25. Time sequence of processed fluorescence microscopy images **(a-e)** showing the propagation of the dewetting front (highlighted via yellow lines) of the multiphase coacervate droplets. All multiphase coacervate droplets are in a complete wetting condition (pH 8.3, red colored droplets) at the start of the experiment. **(f)** The lines showing the position of the reconfiguration front at different times are overlayed on a processed fluorescence microscopy image captured at t = 108 min. Scale bar is 1 mm.

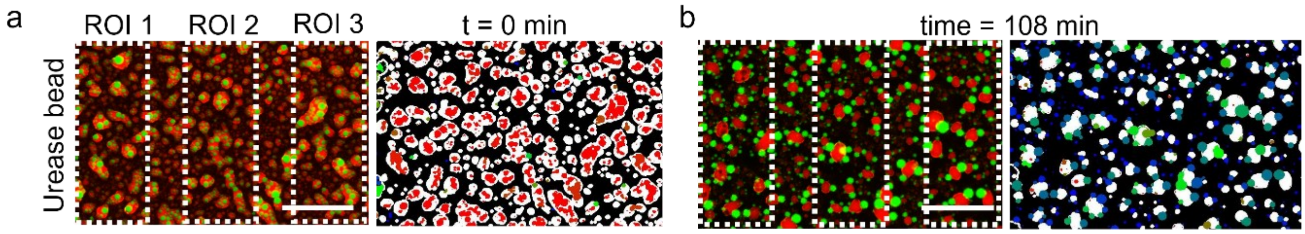

Figure S26. (a-b) Fluorescence microscopy image (left) and MATLAB color coded image (right) of DEAE/PAA/PDDA multiphase coacervate droplets (2.21/40/10 mM) at t = 0 min (a) and t = 108 min (b) with different ROI regions (ROI 1-3, white dashed outlines) identified to track the dewetting front triggered by urease gel beads (placed to the left of the viewing area) from left to right. Scale bars are 100 µm.

References

1 G. A. Mountain and C. D. Keating, *Biomacromolecules*, 2020, **21**, 630–640.

2 W. Mu, Z. Ji, M. Zhou, J. Wu, Y. Lin and Y. Qiao, *Sci. Adv.*, 2021, **7**, 9000–9028.

3 M. Feric, N. Vaidya, T. S. Harmon, D. M. Mitrea, L. Zhu, T. M. Richardson, R. W. Kriwacki, R. V. Pappu and C. P. Brangwynne, *Cell*, 2016, **165**, 1686–1697.

4 R. S. Fisher and S. Elbaum-Garfinkle, *Nat. Commun.*, 2020, **11**, 4628.

5 J. R. Kanicky and D. O. Shah, *J. Colloid Interface Sci.*, 2002, **256**, 201–207.

6 P. S. Patwal, S. Mann and B. V. V. S. P. Kumar, *Adv. Mater.*, 2025, **37**, 2415568.

7 B. Munavirov, O. Gnezdilov, M. Rudakova, O. N. Antzutkin and A. Filippov, *Magn. Reson. Chem.*, 2013, **51**, 750–755.

8 F. De Paula Pansani Oliveira, I. P. D. Picola, Q. Shi, H. F. G. Barbosa, V. A. De Oliveira Tiera, J. C. Fernandes and M. J. Tiera, *Nanotechnology*, 2013, **24**, 055101.

9 H. Y. Ta, C. Déjugnat, S. Balayssac, F. Collin, S. Balor, V. Gilard and F. Couderc, *J. Mol. Liq.*, 2022, **360**, 119478.

10 A. T. Horvath, A. E. Horvath, T. Lindström and L. Wågberg, *Langmuir*, 2008, **24**, 10797–10806.

11 A. B. Kayitmazer, D. Shaw and P. L. Dubin, *Macromolecules*, 2005, **38**, 5198–5204.

12 A. Shakya and J. T. King, *Biophys. J.*, 2018, **115**, 1840–1847.
